# C-terminus-dependent detection of lysosomal alpha-synuclein in nigral Parkinson’s disease human brain neurons

**DOI:** 10.1186/s13024-025-00884-3

**Published:** 2025-10-31

**Authors:** Martino L. Morella, Bana Al Khayrat, Tim E. Moors, Lisanne in’t Veld, Irene Frigerio, Vinod Udayar, Bram L. van der Gaag, Wilma D. J. van de Berg

**Affiliations:** 1 Department of Anatomy & Neurosciences, Chair Section Clinical Neuroanatomy and Biobanking, Amsterdam Neuroscience, Amsterdam UMC, Vrije University Amsterdam, O|2 Life Sciences Building, Room 13 E55, De Boelelaan 1108, Amsterdam, HZ 1081 The Netherlands; 2 Amsterdam Neuroscience, Program Neurodegeneration, Amsterdam, The Netherlands; 3 Roche Pharma Research and Early Development; Neuroscience and Rare Diseases Discovery and Translational Area; Roche Innovation Center, F. Hoffmann-La Roche Ltd, Grenzacherstrasse 124, Basel, 4070 Switzerland

**Keywords:** Alpha-synuclein, Lysosome, C-terminus, Parkinson’s, Truncation, Morphology

## Abstract

The abnormal accumulation of alpha-Synuclein (αSyn) within neurons is a hallmark of synucleinopathies, such as Parkinson's disease (PD), and could stem from impaired protein degradation. Genetic, in vitro, and *post-mortem* studies have suggested that lysosomal dysfunction and impaired proteolytic activity play important roles in the pathogenesis of PD. Lysosomes have been proposed as key sites for αSyn degradation, but direct evidence of the lysosomal localization of endogenous αSyn in the human brain is limited. This study aimed to investigate the localization of αSyn proteoforms, including different post-translational modifications (PTMs), within lysosomes of *post-mortem* human nigral neurons. We analyzed formalin-fixed, paraffin-embedded brain tissue from donors diagnosed with PD, PD with Dementia (PDD) or incidental Lewy body disease (iLBD). *Substantia nigra* sections were assessed using an extensive panel of αSyn-specific antibodies, including PTM-specific antibodies, and selected lysosomal markers via multiplex immunofluorescence, confocal and stimulated emission depletion (STED) microscopy. Here, we demonstrate widespread accumulation of αSyn within lysosomes in nigral dopaminergic neuron somas of donors with PD/PDD and iLBD. This lysosomal αSyn appeared morphologically distinct from cytosolic inclusions such as Lewy bodies (LBs) and related macro-aggregates, and was present both in cells with and without these larger αSyn deposits. When present, macro-aggregates were consistently accompanied by ring-shaped lysosomal structures. Compared to other neuronal morphologies, lysosomal αSyn was the most frequent morphology at early Braak stages (1–4), with a decline at later stages (5–6). Interestingly, lysosomal αSyn was detected solely by targeting the N-terminus or the NAC domain of αSyn, and not with antibodies targeting Serine 129-phosphorylated αSyn or other epitopes at the C-terminus (CT), suggesting that lysosome-associated αSyn lacks the CT. Our findings reveal two co-existing pools of neuronal somatic αSyn: a CT-negative lysosome-associated form, and a primarily non-lysosomal CT-positive form. Overall, we provide direct evidence of lysosomal involvement in cellular αSyn metabolism in *post-mortem* human PD brain.

**Supplementary Information:**

The online version contains supplementary material available at 10.1186/s13024-025-00884-3.

## Introduction

Alpha-synuclein (αSyn), a small and intrinsically disordered protein predominantly expressed in neurons [1, 2], is implicated in several neurodegenerative diseases collectively termed synucleinopathies. These include Parkinson’s disease (PD) and dementia with Lewy bodies, in which αSyn progressively accumulates in specific brain regions [3]. αSyn is a 140-amino-acid protein comprising three distinct domains: an amphipathic N-terminus (NT), a hydrophobic central domain known as the non-amyloid-β component (NAC), and a negatively charged C-terminus (CT) [4, 5]. The NT region (residues 1–60), characterized by its amphipathic nature, is thought to facilitate lipid binding and membrane interactions [6, 7]. The NAC domain (residues 61–95), a hydrophobic region, is implicated in protein aggregation and fibril formation [8–10]. In contrast, the CT domain (residues 96–140) is negatively charged and intrinsically disordered, playing a role in interactions with other proteins and metal ions [11].

Within neurons, aggregated αSyn accumulates in distinct intracellular macro-aggregates, such as Lewy bodies (LBs) and Lewy neurites (LNs), which are hallmark features of synucleinopathies [12]. LNs manifest as thread-like fibrillar deposits along axons and dendrites, whereas LBs are spherical, cytoplasmic inclusions composed of αSyn, along with other cellular components [13, 14]. Recent studies have revealed that mature nigral LBs in PD exhibit a distinctive onion-skin-like architecture [14]. This structured framework consists of Serine 129-phosphorylated αSyn and cytoskeletal elements encapsulating a core enriched with CT-truncated (CTT) αSyn species. Additionally, these LBs are rich in membranous structures, including vesicles, lysosomes, and dysmorphic organelles, with αSyn interspersed among them [13, 14], suggesting that the buildup of disrupted cellular membranes and organelles significantly contributes to the development of Lewy pathology. In addition to LBs and LNs, a spectrum of cytoplasmic macro-aggregates has been described, ranging from small, amorphous inclusions to cytoplasmic reticular networks, possibly representing earlier stages of intracellular pathology formation [14]. Elucidating the temporal sequence of events leading to the formation of macro-aggregates is critical for unraveling the mechanisms underlying cellular dysfunction in synucleinopathies and developing effective therapeutic strategies.

αSyn undergoes various post-translational modifications (PTMs) that critically affect its structure, aggregation propensity, and pathological role in synucleinopathies [15]. Prominent PTMs include phosphorylation, ubiquitination, acetylation, nitration, glycation, and truncation. Among these, phosphorylation at Serine 129 (pSer129) is the most extensively studied and is strongly associated with pathology. In healthy brains, only a small fraction of αSyn is phosphorylated at Serine 129, but this modification is markedly upregulated under pathological conditions [16]. The truncation of αSyn, particularly at its CT domain, has also been shown to influence pathological aggregation and toxicity in synucleinopathies [17, 18]. The CT region of αSyn is rich in acidic residues and plays a critical role in maintaining protein solubility and inhibiting aggregation. Proteolytic cleavage by enzymes such as calpains, cathepsins, or matrix metalloproteinases removes parts of this domain, destabilizing the protein and promoting the formation of aggregation-prone species [19, 20]. Specific CTT αSyn variants, such as those truncated at amino acids 119 (CTT119) and 122 (CTT122), are abundant in the LBs of PD patients brains [16, 21–23], but are also detectable biochemically in healthy brains [24, 25]. Importantly, these CTT forms are not detected by antibodies recognizing pSer129 or the CT region of αSyn, which are commonly used in diagnostic procedures such as Braak staging [26]. Consequently, the prevalence and role of CTT αSyn in disease pathology may currently be underestimated, underscoring the need for comprehensive detection strategies using panels of antibodies against different epitopes.

αSyn has been proposed to be directly degraded by the lysosome [27–29], and its lysosomal localization has been demonstrated in several in vitro and animal models [30–34]. Moreover, αSyn possesses the KFERQ-like motif necessary for lysosomal targeting by chaperone-mediated autophagy (CMA), and has been shown to be specifically degraded by CMA both in vitro and in vivo [29, 35, 36]. Similarly, αSyn has been reported to disrupt vesicular trafficking, which is essential for lysosomal function, thereby inhibiting the cellular response to lysosomal stress and promoting the persistence of aggregates [37, 38]. These results indicate that the lysosome is a central site for αSyn processing and regulation. Nonetheless, direct evidence of endogenous αSyn accumulation at the lysosome in *post-mortem* human PD brain is limited [31, 39]. Investigating this relationship in *post-mortem* PD brains is crucial for understanding the mechanisms underlying αSyn degradation, aggregation and cellular degeneration.

Here, we aimed to investigate the localization of αSyn proteoforms and PTMs in lysosomes of human *post-mortem* nigral neurons from neuropathologically confirmed donors diagnosed with either PD, PDD, or iLBD, as well as in age-matched controls, by confocal and super-resolution stimulated emission depletion (STED) microscopy. We report abundant accumulation of αSyn within lysosomes in dopaminergic (DA) neuron somas of the SN in PD/PDD and iLBD subjects, but not in controls, both in the presence and absence of somatic macro-aggregates (such as LBs). All cells with somatic macro-aggregates also displayed lysosomal αSyn. Cells displaying only lysosomal αSyn were the most frequent morphology at early Braak stages (Braak 1–4), and declined in later Braak stages (Braak 5–6) in the SN. Moreover, lysosomal αSyn was not detected by antibodies targeting the CT, suggesting it may lack the CT domain. Overall, we reveal two distinct intraneuronal pools of pathology-associated αSyn: a CT-negative lysosomal form, and a non-lysosomal form with an intact CT, which is predominantly phosphorylated at Ser129. Our findings indicate lysosomal involvement in the processing of accumulated αSyn in *post-mortem* human PD brain, and underscore the therapeutic potential of targeting lysosomes to mitigate αSyn accumulation and slow disease progression.

## Materials and methods

### Post-mortem human midbrain tissue

*Post-mortem* human brain tissue was obtained from the Netherlands Brain Bank (NBB; www.brainbank.nl) from neuropathologically-verified donors in the following groups: individuals with clinically-diagnosed and neuropathologically confirmed idiopathic PD (*n* = 8) or PDD (*n* = 10) and age-matched control subjects (*n* = 8). In addition, we included individuals with incidental Lewy body disease (iLBD; *n* = 30). Informed consent for brain autopsy, use of brain tissue, and sharing of clinical information for research was obtained from either the donors or their families, adhering to all ethical and legal guidelines. Brain dissections followed standard operating protocols established by the NBB, with neuropathological assessments conducted by a qualified neuropathologist [40, 41].

Donors in the PD/PDD group were selected with minimal Alzheimer's disease pathology (Braak neurofibrillary tangle stage ≤ 3) and the absence of micro-infarcts [42, 43]. iLBD cases had a Braak αSyn stage from 1 to 5 and did not have a clinical diagnosis of PD. Control subjects were selected with no αSyn pathology at neuropathological assessment (Braak αSyn stage = 0) and no history of neurological disorders. Supplementary Table 1 and Supplementary Table 2 (see Additional file 1) provide the demographics and characteristics of the selected groups. The diagnostic groups were matched for age at death, and did not show statistically significant differences in age at disease onset or disease duration between the groups. For microscopy analysis, 20 and 30 μm thick sections were prepared from formalin-fixed paraffin-embedded (FFPE) tissue blocks of the midbrain, specifically containing the SN from each included donor. These sections were then used for immunohistochemistry (IHC) and multiplex immunofluorescence (MxIF) in the various labeling experiments.

### Immunohistochemistry

Following deparaffinization of the sections, antigen retrieval (AR) was conducted by either steaming the sections in a 10 mM citrate buffer (pH 6) at 96ºC for 30 min, or by incubating the section in 100% formic acid for 10 min, or both. The AR used for each antibody staining is specified in Supplementary Table 3 (see Additional file 1). For single-labelling experiments with colorimetric detection, the 20 μm thick FFPE sections were incubated for 30 min in 1% H_2_O_2_ in TBS to block endogenous peroxidase activity. The sections were then blocked for 1 h in a blocking buffer (BB1) containing 2% normal donkey serum (#017–000-121, Jackson Immunoresearch) and 0.1% Triton-X in TBS (pH 7.6). Primary antibodies were then applied to the sections, which were incubated overnight at 4ºC.

#### Colorimetric detection

The sections were washed in TBS and incubated with mouse Envision + HRP solution (Agilent-Dako, # K4001) for 35 min to fully develop the signal. The sections were then washed twice in TBS and once in PBS for 5 min and incubated in Vector SG HRP Substrate solution (Vector SG HRP Substrate kit, Vector Laboratories, #SK-4700) for 10 min. After, the sections were washed twice in PBS for 5 min, incubated for 1 min in Nuclear Fast Red Counterstain solution (Vector Laboratories, # H-3403–500), and washed for 5 min under running tap water. Finally, the slides were submerged in increasing ethanol solutions (70%, 80%, 96%, 2 × 100% ethanol in water), 3 times in xylene solution, and mounted in Entellan mounting media (Sigma-Aldrich, #107,960) with a glass coverslip.The sections were washed in TBS and incubated with mouse Envision + HRP solution (Agilent-Dako, # K4001) for 35 min to fully develop the signal. The sections were then washed twice in TBS and once in PBS for 5 min and incubated in Vector SG HRP Substrate solution (Vector SG HRP Substrate kit, Vector Laboratories, #SK-4700) for 10 min. After, the sections were washed twice in PBS for 5 min, incubated for 1 min in Nuclear Fast Red Counterstain solution (Vector Laboratories, # H-3403–500), and washed for 5 min under running tap water. Finally, the slides were submerged in increasing ethanol solutions (70%, 80%, 96%, 2 × 100% ethanol in water), 3 times in xylene solution, and mounted in Entellan mounting media (Sigma-Aldrich, #107,960) with a glass coverslip.

#### Multiple immunofluorescent detection

Multiple-labeling experiments (up to 4 fluorescent labels) were conducted on either 20 or 30 μm thick FFPE sections. The sections were initially washed and incubated with the appropriate secondary antibodies diluted in BB1 for 2 h at room temperature. For labelling experiments involving the use of two antibodies from the same species, after secondary antibody incubation the sections were blocked for 1 h in BB2 (a solution containing 5% normal rabbit serum (#011–000-120, Jackson Immunoresearch) and 5% normal mouse serum (#015–000-120, Jackson Immunoresearch) in TBS). The samples were then incubated for an additional 2 h at room temperature in BB2 containing biotinylated versions of the antibody of interest followed by incubation with Alexa Fluor 546-labelled streptavidin (#S11225, Thermo Fisher Scientific). Details on the primary and secondary antibodies used in this study and their concentrations are presented in Supplementary Table 3 and Supplementary Table 4 (see Additional file 1). Details on the αSyn antibody epitopes and related publications are presented in Table 1. In the final incubation step of all slides, DAPI was added to the buffer at a concentration of 2 µg/ml. Sections were then washed and mounted using Mowiol mounting solution containing 2.5% 1,4-diazobicyclo-[2.2.2]-octane (DABCO 33-LV, #290,734, Sigma-Aldrich) and glass coverslips (#630–2746; Glaswarenfabrik Karl Hecht, Sondheim, Germany). Stainings without primary antibodies were performed as negative controls to assess background, autofluorescence levels and nonspecific staining. Additionally, single labeling for each antibody used in multiple-labeling experiments was carefully examined to ensure that the immunoreactivity patterns were not due to cross-reactivity between antibodies.

**Table 1:** Details of the αSyn antibodies used in the present study

Clone Name	Reference nr	RRID	Source	Epitope	Domain	Host (clonality)	Reference
A15110D (LASH-BL 34–45)	849102	AB_2650701	Biolegend	aa 34–45	N-terminus	Mouse (monoclonal)	[44]
A15119B (LASH-BL pY39)	849201	AB_2650704	Biolegend	pTyr39	N-terminus	Mouse (monoclonal)	[45]
5G4	MABN389	AB_2716647	Merck Millipore	aa 46–53	N-terminus	Mouse (monoclonal)	[46]
A15115A	848302	AB_2650688	Biolegend	aa 80–96	NAC	Mouse (monoclonal)	[46]
Syn1 (Clone 42)	610786	AB_398107	BD Biosciences	aa 91–99	NAC(91–95)/C-terminus(96–99)	Mouse (monoclonal)	[47]
4B12	807801	AB_2564730	Biolegend	aa 103–108	C-terminus	Mouse (monoclonal)	Producer
LB509	ab27766	AB_727020	Abcam	aa 115–122	C-terminus	Mouse (monoclonal)	[48]
5C1	n.a	n.a	Prothena (gift)	aa 118–126	C-terminus	Mouse (monoclonal)	[49]
MJFR1	ab209420	AB_2537217	Abcam	aa 118–123	C-terminus	Rabbit (polyclonal)	Producer
asyn-131	n.a	n.a	Roche (gift)	CTT119	C-terminus	Rabbit (polyclonal)	[14]
Syn105	n.a	n.a	Prothena (gift)	CTT122	C-terminus	Rabbit (polyclonal)	[50]
A15127A	848402	AB_2650690	Biolegend	CTT122	C-terminus	Mouse (monoclonal)	[45]
11A5	n.a	n.a	Prothena (gift)	pSer129	C-terminus	Mouse (monoclonal)	[14, 16]
EP1536Y	ab51253	AB_1193226	Abcam	pSer129	C-terminus	Rabbit (polyclonal)	[51, 52]

### High-content slide imaging and quantification

High-magnification images of the whole SN area of the single-labelling colorimetric IHC stainings were acquired using a whole-slide scanner (Olympus VS200 Evident, 60 × UPLXAPO60XO 1.42 NA oil objective) after manual segmentation of the SN based on 4 × overview scans. Seven planes were acquired over a z-stack of 18 µm (z-step = 3 µm) in the center of the 20 µm slide using Extended focal imaging with a 50% z-plane distribution relative to the focal plane (SLIDEVIEW VS200 ASW software, v3.3). Image processing was performed in QuPath 0.2.3 [53] and FIJI (ImageJ2, National Institutes of Health, USA; [54]). The Vector SG (αSyn)-positive and neuromelanin-positive area was quantified with in-house QuPath scripts using consistent intensity thresholding and size exclusion criteria for all samples, and was expressed as percentage of the total area based on the SN segmentation area. The quantification of αSyn morphologies was performed manually on anonymized images in QuPath after manual drawing of a region of interest (ROI) around the boundaries of the SN. Categorization tables for this quantification are presented in Supplementary Table 5 and Supplementary Table 6 (see Supplementary file 1).

### Confocal and stimulated emission depletion imaging

CSLM and STED microscopy were conducted using a Leica TCS SP8 STED 3X gated microscope (Leica Microsystems). A 60 × 1.4 NA oil immersion lens was employed for confocal imaging, while a HC PL APO CS2 100 × 1.4 NA oil lens was used for STED imaging. Both the zoom and resolution were optimized to achieve a theoretical pixel size below 15 nm × 15 nm. Images were captured in counting mode using gated hybrid detectors, with sequential scanning for each fluorophore by irradiating with a pulsed white light laser at specific wavelengths. For STED imaging, a pulsed 775 nm STED laser line was applied to deplete the Abberior STAR 635P fluorophore (#ST635P-1002, Abberior), and a continuous wave (CW) STED laser at 592 nm was used for depletion of the Alexa Fluor 488 fluorophore (#A-21202, Thermo Fisher Scientific).

Following image acquisition, deconvolution was performed using CMLE (for confocal) or GMLE (for STED) algorithms in Huygens Professional software (Scientific Volume Imaging, Huygens, Netherlands). The acquisition settings, including exposure and gamma, were held constant for all images, with final brightness and contrast adjustments performed in ImageJ (National Institutes of Health, USA). Images of neuromelanin-containing cells were obtained within the SN, focusing on cells with a discernible nucleus and defined cytoplasmic area in the same z-plane. A comprehensive manual review across sections was performed, with a representative z-stack scanned at 0.9 µm, using a z-step of 0.15 µm, inclusive of the nucleus. For 3D reconstruction, a minimum of 18 planes were scanned with a z-step of 0.20 µm. Cells were selected for imaging based on the presence of neuromelanin within soma, which was assessed prior confocal scanning by bright-field. Over 150 neuromelanin-containing dopaminergic neurons, including those from control subjects, iLBD cases, and PD/PDD patients, were imaged. The full range of observed morphologies is presented throughout the manuscript.

### Quantification of 3D surface colocalization

To quantify lysosome-localized αSyn, we generated 3D surface renderings using Imaris (Oxford Instruments, v10.2.0) for both lysosomal and αSyn channels. The 3D surfaces were rendered based on consistent absolute intensity thresholding values and filtering criteria throughout the dataset. Signal colocalization was measured as colocalized surface using the Surface-Surface Colocalization extension (Imaris), which identified overlapping voxels between two surface objects. The results are expressed as relative percentage of αSyn surface colocalized with the lysosomal surface.

### Statistics and computing

The data were analyzed and graphed using R (R version 4.3.2, [55]2023–10-31 [56]) and R Studio [55]. Results were averaged for each subject and, for normally distributed data, one-way or two-way ANOVA followed by Bonferroni-corrected pairwise t-tests was performed. For non-normally distributed data, the Kruskal–Wallis test was conducted, followed by Bonferroni-corrected Wilcoxon Rank-Sum tests for pairwise comparisons, when applicable. When comparing neuromelanin-positive area and morphological counts across Braak stages, a generalized linear model (GLM) with a Gamma distribution and log link function was used, adjusting for age, sex and *post-mortem* delay. A constant of 0.0001 was added to zero values to enable modeling. Pairwise comparisons between Braak stages were performed using Tukey-adjusted estimated marginal means. Correlation analysis was performed with Spearman’s correlation analysis. Spearman correlation coefficient (ρ) is reported. In all tests, significance threshold was set at *p* ≤ 0.05. The statistics used in each analysis is reported in the figure legends. The graphical summary (Fig. 7) was created using Biorender (BioRender.com, 2024).

## Results

### Non-phosphorylated Ser129 αSynuclein displays ring-shaped organellar morphology in Parkinson’s disease nigral dopaminergic neurons

**Fig. 1:**
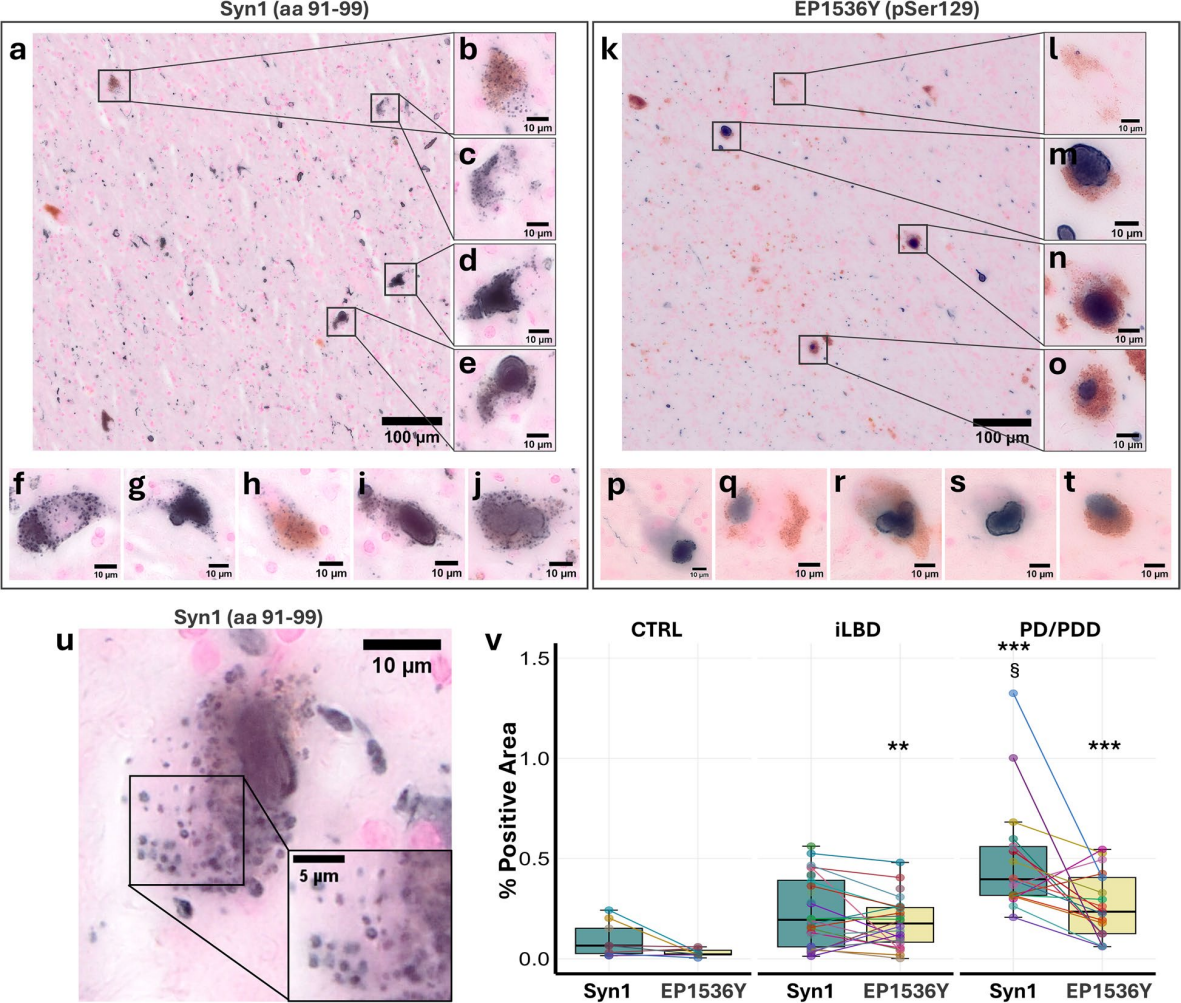
Ser129-unphosphorylated αSynuclein displays ring-shaped organellar morphology in human PD/PDD *substantia nigra* neurons — **a-u** Representative immunohistochemistry images of somatic αSynuclein (αSyn) morphologies (blue staining) in dopaminergic *substantia nigra* (SN) neurons observed in Parkinson’s disease (PD) and PD with dementia (PDD) *post-mortem* human brain. **a-j** Immunostaining with Syn1 antibody (αSyn aa 91–99). Lower magnification images (**a**) identify abundant somatic and extrasomatic positively stained areas. Higher magnifications (**b-j**) of neuromelanin-positive neuronal somas show the existence of a variety of αSyn morphologies, including ring-shaped and dotted-like morphologies, with (**d-g**,**i,j**) and without (**b**,**c**,**h**) amorphous macro-aggregates or Lewy body (LB)-like morphologies (**e**). **k-t** Immunostaining with EP1536Y antibody (Serine 129-phosphorylated αSyn, pSer129). Lower magnification images (**k**) identify abundant somatic and extrasomatic positively stained areas, but to a lesser extent compared to what is observed with Syn1 antibody. Somatic pSer129 αSyn neuronal cytopathology (**l–t**) includes diffuse cytoplasmic staining both with (**m-p**, **r**, **s**) and without (**q**, **t**) larger amorphous aggregates and LB-like morphologies, but not dotted like-morphologies. **u** Close-up of a deconvolved high-resolution representative Syn1 IHC image of a PD neuron from the SN showing the ring-shaped and organellar appearance of dotted-like αSyn cytopathology (yellow arrows). **v** Quantification of the percentage of αSyn-positive IHC area in the SN of control (CTRL), incidental Lewy body disease (iLBD) and PD/PDD cases. ** *p* < 0.01 vs CTRL; *** *p* < 0.005 vs CTRL; § *p* < 0.05 vs iLBD (Kruskal–Wallis + Bonferroni-corrected Wilcoxon rank-sum post-hoc test). aa = amino acid

To investigate the subcellular manifestations of αSyn in nigral dopaminergic neurons, we assessed the immunoreactivity patterns of αSyn using an antibody with epitope between amino acids (aa) 91 and 99 (between the NAC and CT regions; clone Syn1). Following formic acid and citrate buffer heat-induced epitope retrieval pre-treatment, negligible staining was observed in the control cases (Suppl. Figure 1, see Additional file 1), while in PD(D) and iLBD, Syn1 staining revealed a range of morphologies in neuronal somas, as well as extrasomatic profiles such as dotted-like αSyn aggregates and (dystrophic) LNs (Fig. 1a-j; Suppl. Figure 1, see Additional file 1) across all PD/PDD and iLBD cases. This variability was also apparent among neurons within the same individual. Non-somatic morphologies included the presence of dotted αSyn staining and a variety of LN-like morphologies, both with and without additional swelling (bulgy neurites) (Fig. 1a; Suppl. Figure 1, see Additional file 1). Most notably, in addition to different perinuclear inclusions, neuronal somatic depositions included various degrees of dotted staining throughout the cytoplasm. These patterns were common both in cells without (Fig. 1b,h) and with (Fig. 1c-g,i,j) cytoplasmic macro-aggregates. Intracellular staining was observed throughout the neuronal cytoplasm but never in the nucleus. Importantly, the number, size, and intensity of the dotted staining varied both between and within cases (Fig. 1a-j; Suppl. Figure 1, see Additional file 1). Even within single cells, individual puncta differed in size and intensity. In a variable fraction of neurons, the dotted staining was accompanied by the presence of macro-aggregates of various sizes. This included small (Fig. 1c), medium (Fig. 1f) and large (Fig. 1d,g,l,j) amorphous aggregates, as well as fully-formed intracellular LBs (Fig. 1e). Interestingly, we identified neurons with dotted morphology and no macro-aggregates, whereas no neurons containing only macro-aggregates and no dotted morphologies could be observed. When studying the dotted morphology at higher magnification, the staining showed a vesicular ring-shaped morphology with a darker staining at the periphery of each punctum and a diffuse staining in the center (Fig. 1u).

Interestingly, these neuronal somatic dotted patterns were not observed when using an antibody against pSer129 αSyn (clone EP1536Y; Fig. 1k-t). Instead, only small (Fig. 1l) and large (Fig. 1m-p,r,s) macro-aggregates, and a faint diffuse cytoplasmic staining (Fig. 1p-t), could be identified in addition to extrasomatic aggregates, as previously described [14]. In addition to the neuronal morphologies, extrasomatic dotted αSyn staining and a variety of LN morphologies could be observed, as before, but to a lesser extent (Fig. 1k; Suppl. Figure 1, see Additional file 1). Quantification of the percentage of αSyn-positive area normalized to total SN area confirmed the increased immunoreactivity in PD/PDD compared to controls, with Syn1 staining showing the highest increase (Fig. 1v). When comparing the two stainings in adjacent sections in the same case, Syn1 staining could identify a higher percentage of αSyn-positive area, suggesting this antibody can stain additional morphology types compared to pSer129 αSyn staining, in line with previous findings [31, 44].

### Progressive shift in the ratio of somatic αSynuclein morphologies across Braak stages in substantia nigra neurons

**Fig. 2:**
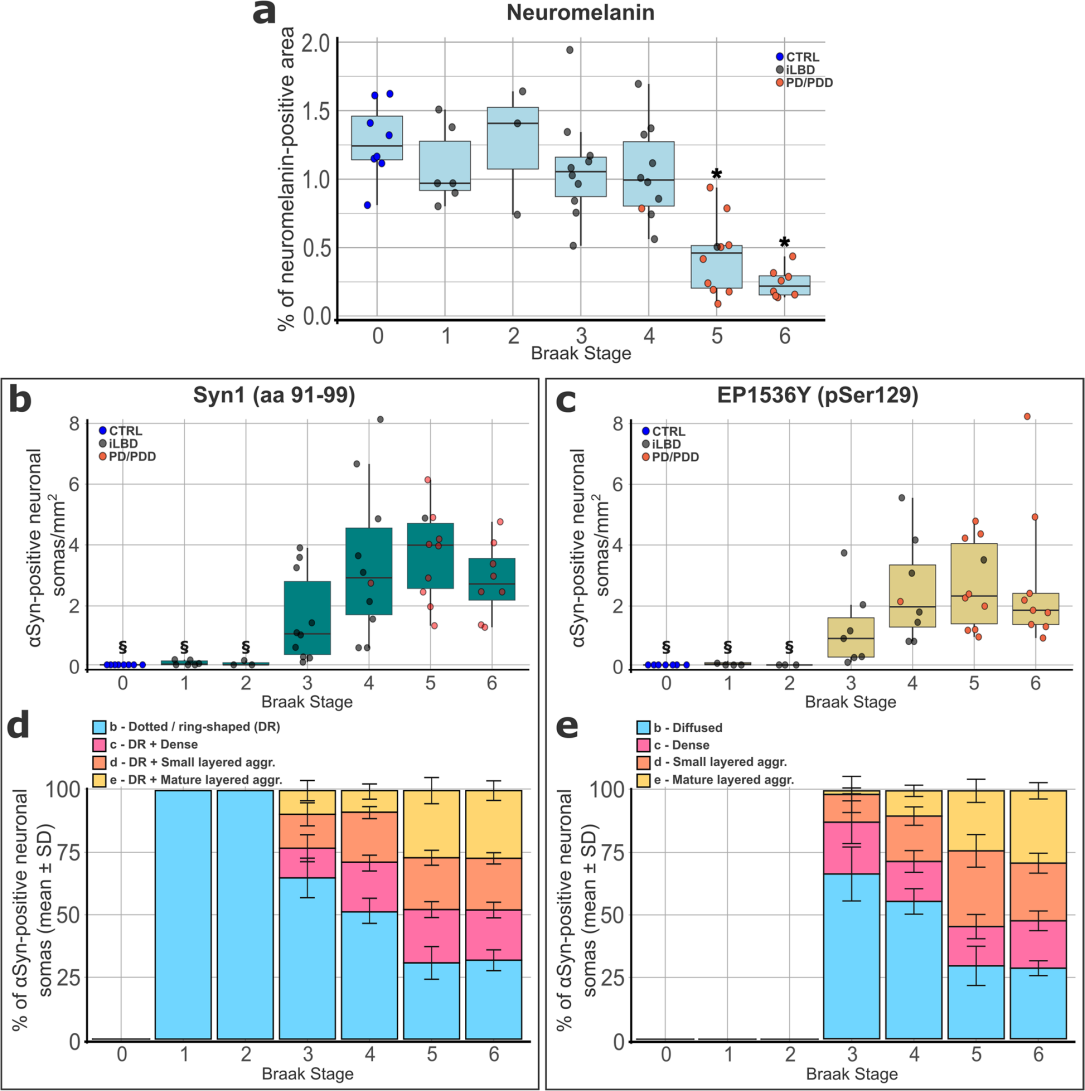
Quantification of somatic Syn1 and pSer129 αSynuclein morphologies in *substantia nigra* PD/PDD and iLBD neurons — Quantification of neuromelanin and αSynuclein (αSyn) somatic morphologies in Parkinson’s disease (PD) and PD with dementia (PDD; PD/PDD), incidental Lewy body disease (iLBD), and control (CTRL) *post-mortem* human brain *substantia nigra* (SN) tissue sections by IHC. **a** Quantification of neuromelanin-positive area percentage. **b-e** Quantification of neuronal somatic αSyn morphologies in tissue stained with either the NAC-targeting antibody Syn1 (aa 91–99; **b,d**) or EP1536Y (Serine 129-phosphorylated αSyn, pSer129; **c,e**). Results are presented for each Braak αSyn stage. **b,c** Total number of αSyn-positive neuronal somas per mm^2^ of SN area. **d,e** Quantification of somatic neuronal αSyn morphologies (as defined in Suppl. Table 5–6; see Additional file 1) expressed as percentage of each morphology relative to the total number of αSyn-positive neuronal somas. a-c: Generalized Linear Model (Gamma distribution; log link) adjusted for age, sex, *post-mortem* delay + pairwise Tukey-adjusted estimated marginal means; * *p* < 0.01 vs Braak 0,1,2,3,4; § *p* < 0.01 vs Braak 3,4,5,6. aa = amino acid

All investigated neurons containing macro-aggregates also exhibited a dotted/ring-shaped morphology. Additionally, we observed neurons with only dotted/ring-shaped αSyn and without macro-aggregates. Therefore, we hypothesized that the proportion of neurons with only the dotted morphology relative to those also containing macro-aggregates varies across Braak stages. To investigate this, we performed a semi-quantitative analysis of αSyn morphologies in SN tissues from PD/PDD (*n* = 18), iLBD (*n* = 30), and control (*n* = 8) cases ranging from Braak stage 0 to 6. Additionally, we quantified the neuromelanin-positive area as a proxy for neuronal loss. The tissue was stained with either Syn1 (aa 91–99) or EP1536Y (pSer129) αSyn-targeting antibodies and imaged using high-content bright-field imaging to quantify morphological features across the entire SN area of a section (Fig. 2). Neuronal somatic αSyn morphologies were defined in five categories: *a*—αSyn-negative; *b*—dotted/ring-shaped (Syn1 staining) or diffused cytosolic (pSer129 staining); *c*—dense inclusion; *d*—small layered aggregate; *e*—mature layered aggregate. In the Syn1 staining, the *c,d,e* categories were always accompanied by additional dotted/ring-shaped αSyn. Details and representative images of each category are presented in Supplementary Table 5 and Supplementary Table 6 (see Additional file 1).

As expected, we observed a reduction in neuromelanin-positive area associated with increased Braak stage (ρ = −0.73, *p* < 0.001; Fig. 2a). The count of total αSyn-positive somatic morphologies per SN area revealed a significant increase in PD/PDD and iLBD cases with Braak stages 3–6 compared to control cases (Braak stage 0) and cases with Braak 1–2 (Syn1: ρ = 0.81, *p* < 0.001; pSer129: ρ = 0.74, *p* < 0.001; Fig. 2b,c). Syn1-based quantification identified a higher total number of αSyn-positive cells compared to pSer129 staining (Fig. 2b,c). Analysis of the frequency of somatic αSyn morphologies (categories *b-e*) between Braak stages revealed that, at lower Braak stages (1–4), the predominant profiles were either dotted/ring-shaped (category *b*, Syn1 staining) or diffused (category *b*, pSer129 staining), together accounting for approximately 50–70% of αSyn-positive neurons (Fig. 2d,e). By contrast, dense inclusions and macro-aggregates (categories *c-e*) comprised only a minor fraction of somatic profiles. Interestingly, dotted/ring-shaped profiles were sporadically observed in sections of Braak stage 1–2 cases (11 cells in 9 cases; 0–2 cells per case), which lacked other somatic morphologies (categories *c-e*). In contrast, pSer129-positive cells were only observed in donors with Braak stage ≥ 4. In the later stages (5–6), we observed a decrease in the fraction of dotted/ring-shaped and diffused morphologies (morphologies *b*) and an increase in the overall proportion of dense and layered macro-aggregates (morphologies *c-e*), both with Syn1 and pSer129 staining, similar to our previous results in a different cohort [14]. Overall, these findings suggest that cytoplasmic diffused αSyn, as identified by pSer129 staining, and particularly the dotted/ring-shaped αSyn, may represent early stages of neuronal somatic αSyn deposition in SN dopaminergic neurons, preceding the formation of macro-aggregates. However, further mechanistic studies are required to confirm this hypothesis.

### Ring-shaped neuronal αSynuclein is localized at the lysosome

**Fig. 3:**
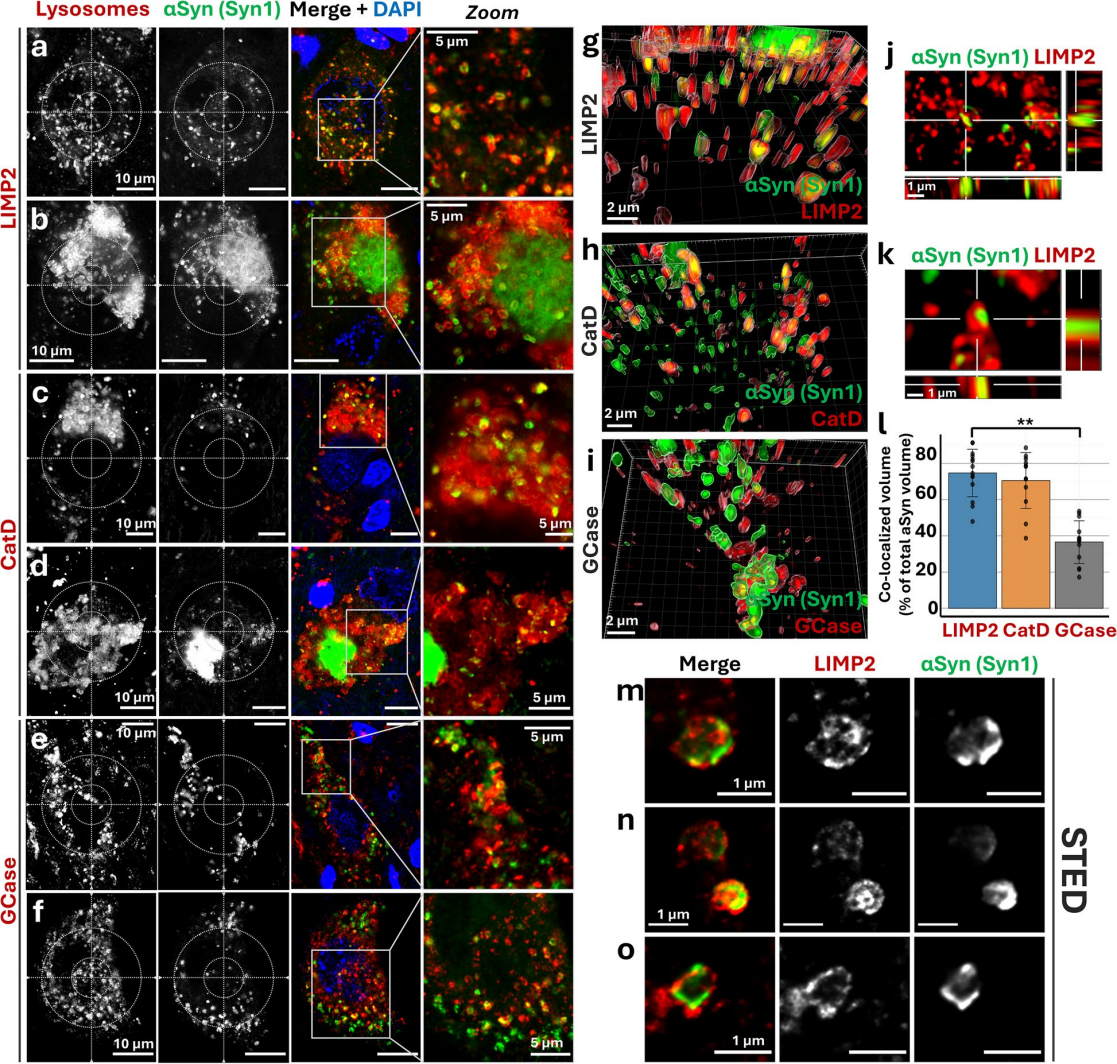
Ring-shaped αSynuclein localizes at the lysosomal pool in human dopaminergic *substantia nigra* neurons — **a-f** Representative deconvolved confocal multiplex immunofluorescence images of αSynuclein (αSyn) (green; Syn1, aa 91–99) in nigral dopaminergic neurons in Parkinson’s disease (PD) and PD with dementia (PDD) *post-mortem* human brain showing the localization of ring-shaped somatic αSyn morphology with lysosomal markers (red throughout). **a-b** Colocalization with LIMP2. **c-d** Colocalization with Cathepsin D (CatD). **e–f** Colocalization with β-Glucocerebrosidase (GCase). **g-k** 3D surface rendering of the indicated stainings (**g-i**) and orthogonal views of representative neuronal lysosomes (**j-k**) showing the localization of αSyn within the lysosomal surface. **l** Single-cell quantification of the percentage of αSyn-positive 3D surface colocalized with the lysosomal surface averaged per PD/PDD case in dopaminergic *substantia nigra* neurons for the indicated lysosomal markers (n ≥ 9; ≥ 6 cells per case; Mean ± SD; ** *p* < 0.01; one-way ANOVA + Tukey's HSD). **m–o** Deconvolved stimulated emission depletion microscopy (STED) images of representative lysosomes (red, LIMP2) showing the localization of αSyn (green, Syn1 aa 91–99) at the lysosome. DAPI is used for visualization of the nuclei (blue). aa = amino acid

Based on the vesicular morphology and its widespread distribution throughout the perikaryon and proximal neurites, we hypothesized that the ring-shaped αSyn signal observed with Syn1 could overlap with lysosomal staining. To test this, we investigated the colocalization between Syn1 and lysosomal markers, such as lysosomal integral membrane protein 2 (LIMP2; Fig. 3a-b,g,j,k; Suppl. Figure 2a-d, see Additional file 1), Cathepsin D (CatD; Fig. 3c-d,h; Suppl. Figure 2e,f, see Additional file 1), and β-Glucocerebrosidase (GCase; Fig. 3e-f,i; Suppl. Figure 2g,h, see Additional file 1). Notably, the staining results demonstrated the colocalization of dotted αSyn with all the lysosomal markers studied. When using GCase as the lysosomal marker, the degree of colocalization appeared lower than with LIMP2 and CatD. Quantification of 3D surface reconstructions showed that the colocalization of GCase and αSyn was lower than that of the other two markers (approximately 50% reduction, Fig. 3l). Moreover, the fluorescent staining revealed different lysosomal populations at this resolution, including large ring-shaped organelles and smaller dotted structures. Their relative abundance varied between cells and was reflected in the αSyn staining pattern. Both ring-shaped and dotted αSyn structures colocalized with lysosomes. However, at higher magnification, the αSyn and lysosomal signals were often in close proximity but not consistently overlapping. Interestingly, 3D surface rendering indicated that the αSyn signal was localized within the reconstructed lysosomal surface defined by the three lysosomal markers (Fig. 3g-l; Suppl. video 1–4, see Additional file 2–5). This was confirmed by super-resolution STED microscopy analysis, which demonstrated that lysosomal αSyn is mostly associated with the lumen or adjacent to the luminal side of the lysosomal membrane surface. Only occasional contact points were observed with the membrane, as identified by the marker LIMP2 (Fig. 3m-o).

### Selective localization of post-translationally modified αSynuclein proteoforms to lysosomes

**Fig. 4:**
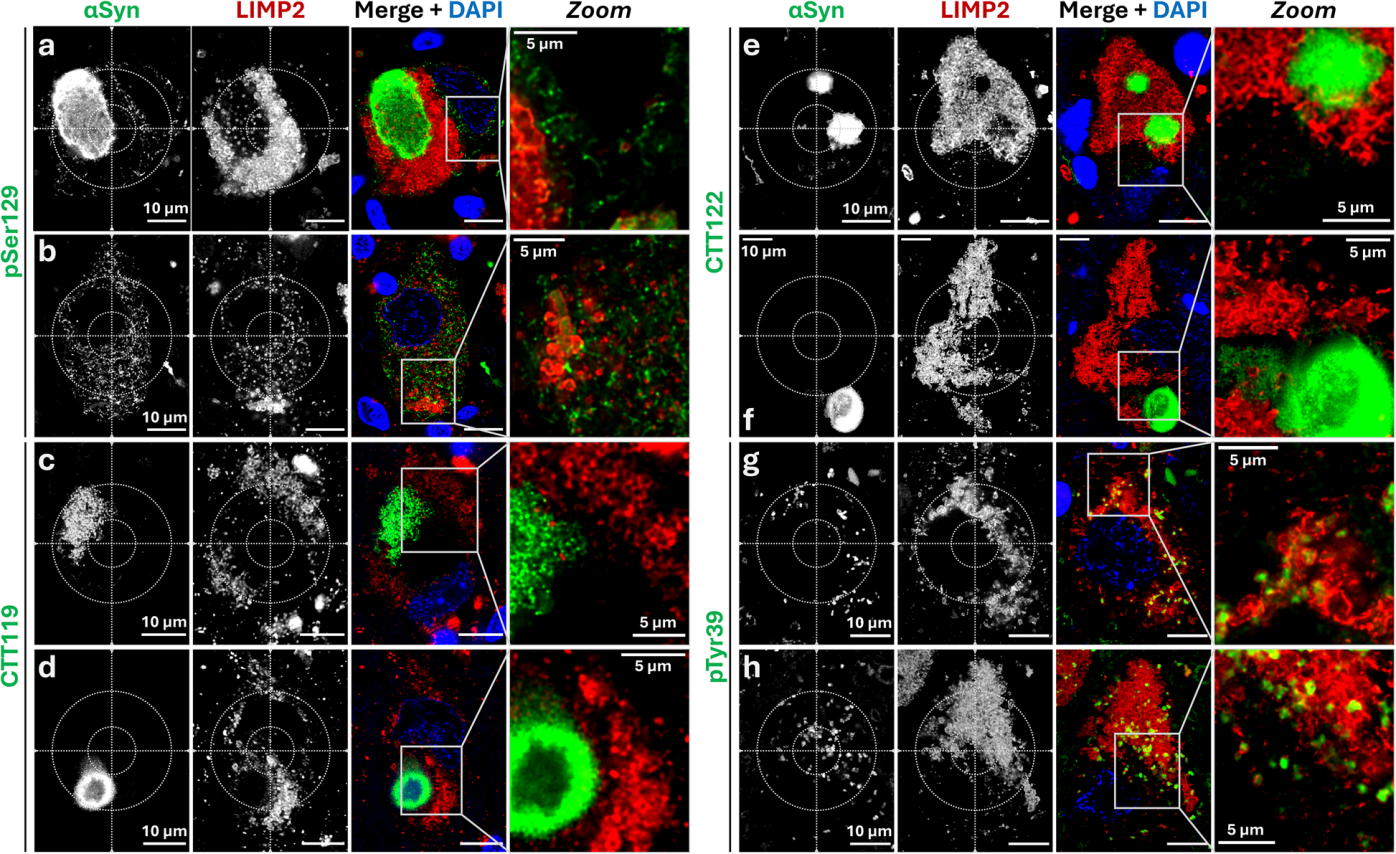
C-terminally modified αSynuclein does not localize at the lysosome in human *substantia nigra* neurons — **a-h** Representative deconvolved confocal multiplex immunofluorescence images and magnifications (right panels, *Zoom*) of dopaminergic neuron somas in Parkinson’s disease (PD) and PD with dementia (PDD) *post-mortem* human *substantia nigra* tissue, stained for the lysosomal marker LIMP2 (red) and antibodies against selected αSynuclein (αSyn) post-translational modifications (green). **a-b** Staining for phosphorylated Serine 129 αSyn (pSer129, clone 11A5) showing limited (**a, b**) colocalization between pSer129 αSyn and the lysosomal pool. **c-f** Staining for C-terminus truncations (CTT) at aa 119 (CTT119, **c-d**) and aa 122 (CTT122, **e–f**), only identifying macro-aggregates and Lewy bodies, and showing no colocalization between the lysosomal pool and truncated αSyn. **g-h** Staining for αSyn Tyr39-phosphorylated (pTyr39) revealing ring-shaped somatic morphologies, which colocalized with the lysosomal marker LIMP2. DAPI is used for visualization of the nuclei (blue). aa = amino acid

Since ring-shaped αSyn was detected with Syn1 (aa 91–99) but not with EP1536Y (pSer129), we investigated whether other post-translationally modified forms of αSyn colocalize with lysosomes. Therefore, we conducted additional LIMP2-αSyn colabelings using PTM-specific antibodies, including pSer129 antibody (clone 11A5; Fig. 4a,b), and antibodies targeting αSyn species truncated at aa 119 (CTT119; Fig. 4c,d) or at aa 122 (CTT122, Fig. 4e,f). We further included an antibody against a NT PTM: αSyn phosphorylated at tyrosine 39 (pTyr39; Fig. 4g,h). The multiplex immunofluorescence staining identified all macro-aggregates, LB somatic morphologies, and cytosolic reticular network patterns. The latter displayed a net-like staining throughout the cytosol and very limited colocalization with lysosomes (Fig. 4a,b), consistent with previous observations [14]. Some degree of colocalization was observed within macro-aggregates, with lysosomal signals appearing inside small amorphous aggregates (Fig. 4b) or within the boundaries of larger aggregates (Fig. 4a), as well as at occasional lysosomal contact points, as previously described [57]. In contrast, both the CTT truncations studied (CTT119, Fig. 4c,d; CTT122, Fig. 4e,f) only recognized LBs and macro-aggregates, but not dotted-like αSyn nor the cytosolic non-lysosomal reticular network observed with pSer129 αSyn-targeting antibodies (Fig. 4c-f). No colocalization with LIMP2 was observed for CTT119 and CTT122, except occasionally with lysosomes present within the macro-aggregates (Fig. 4e). Interestingly, the antibody against the NT PTM pTyr39 showed dotted-like somatic staining patterns similar to those observed with Syn1 (Fig. 4g,h), and these structures colocalized with LIMP2-positive lysosomes.

### Lysosomal αSynuclein lacks the C-terminus, while non-lysosomal αSynuclein is mainly detected by antibodies targeting the C-terminus

**Fig. 5:**
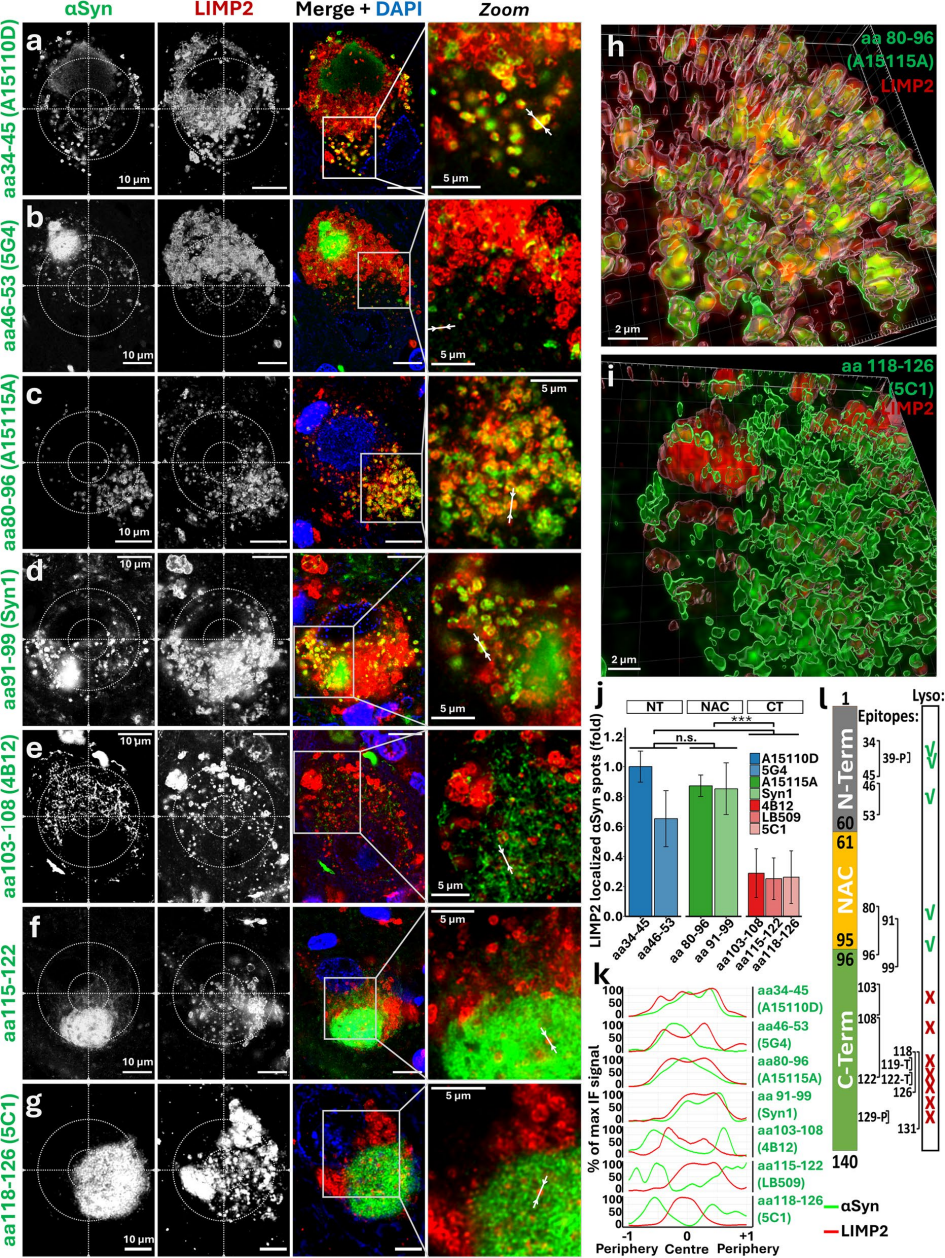
Lysosome-localized αSynuclein proteoforms lack the C-terminus portion in human *substantia nigra* neurons — **a-g** Representative deconvolved confocal multiplex immunofluorescence images and zoom-ins (right panels, *Zoom*) of dopaminergic *substantia nigra* neurons in Parkinson’s disease (PD) and PD with dementia (PDD) *post-mortem* human brain stained for the lysosomal marker LIMP2 (red) and with antibodies against various epitopes along the length of the αSynuclein (αSyn) protein (green), as indicated. Antibodies recognizing epitopes in the N-terminus (NT) or NAC regions (aa 1–99) identify lysosomal-localized ring-shaped αSyn morphology. This morphology is not recognized by antibodies targeting epitopes in the C-terminus (CT) part of the protein, which show limited colocalization with the lysosomal marker. **h-i** 3D surface rendering of representative stainings of LIMP2 (red) and αSyn antibody (green). **h** Staining with αSyn antibody against aa 80–96 (clone A15115A). **i** Staining with αSyn antibody against aa 118–126 (clone 5C1). **j** Single-cell 3D lysosomal αSyn colocalization analysis expressed as percentage of LIMP2-localized αSyn spots per PD/PDD case (folds of aa 34–45; *n* ≥ 7; ≥ 5 cells per case; Mean ± SD; *** *p* < 0.001; 2-way ANOVA (protein domain and antibody as fixed factors) + Bonferroni-corrected t-tests). **k** Line intensity plots of representative LIMP2-positive organelles from the indicated stainings. **l** Graphical summary of the αSyn epitopes and their resulting lysosomal localization, as investigated in this study. Lyso = Lysosomal localization. DAPI is used for visualization of the nuclei (blue). aa = amino acid

Since all αSyn antibodies targeting PTMs at the CT showed limited lysosomal colocalization, unlike Syn1 and pTyr39 NT antibodies, we hypothesize that lysosomal αSyn may lack (part of) the CT, for example due to truncation. Using a panel of αSyn antibodies targeting epitopes along the entire protein sequence (Table 1, Fig. 5), we found that all antibodies targeting the NT and the NAC regions, until clone Syn1 (aa 91–99), showed similar dotted, LIMP2-positive profiles (Fig. 5a-d). In contrast, all αSyn antibodies targeting the CT, starting from antibody clone 4B12 (aa 103–108), identified macro-aggregates and, in some cases, cytosolic reticular networks not associated with lysosomes, similar to those described for pSer129 αSyn (and in previous reports [14]). However, none of the CT antibodies showed ring-shaped staining (Fig. 5e-g, Suppl. Figure 3). Identical morphologies and the same CT-dependent differential staining pattern were observed in noradrenergic neurons of the locus coeruleus in iLBD and PD/PDD cases (Suppl. Figure 4). These observations were further supported by 3D surface renderings, which confirmed the localization of ring-shaped αSyn within the reconstructed lysosomal volume when using NT- and NAC-targeting antibodies (Fig. 5h). CT-targeting antibodies did not show αSyn signal surrounded by reconstructed lysosomal surface, even at contact points (Fig. 5i). Consistently, 3D colocalization analysis across dopaminergic neuronal somas showed reduced lysosomal localization of αSyn when detected with CT-targeting antibodies, as measured by the percentage of LIMP2-localized αSyn spots (Fig. 5j). Line intensity plots of LIMP2-positive organelles confirmed that this change in lysosomal localization was dependent on the targeting of the CT epitope, even when analyzing lysosomes within αSyn macro-aggregates (Fig. 5k). Overall, these results indicate that the main lysosomal αSyn proteoform lacks part of the CT, at least from aa 107 (Fig. 5l).

**Fig. 6:**
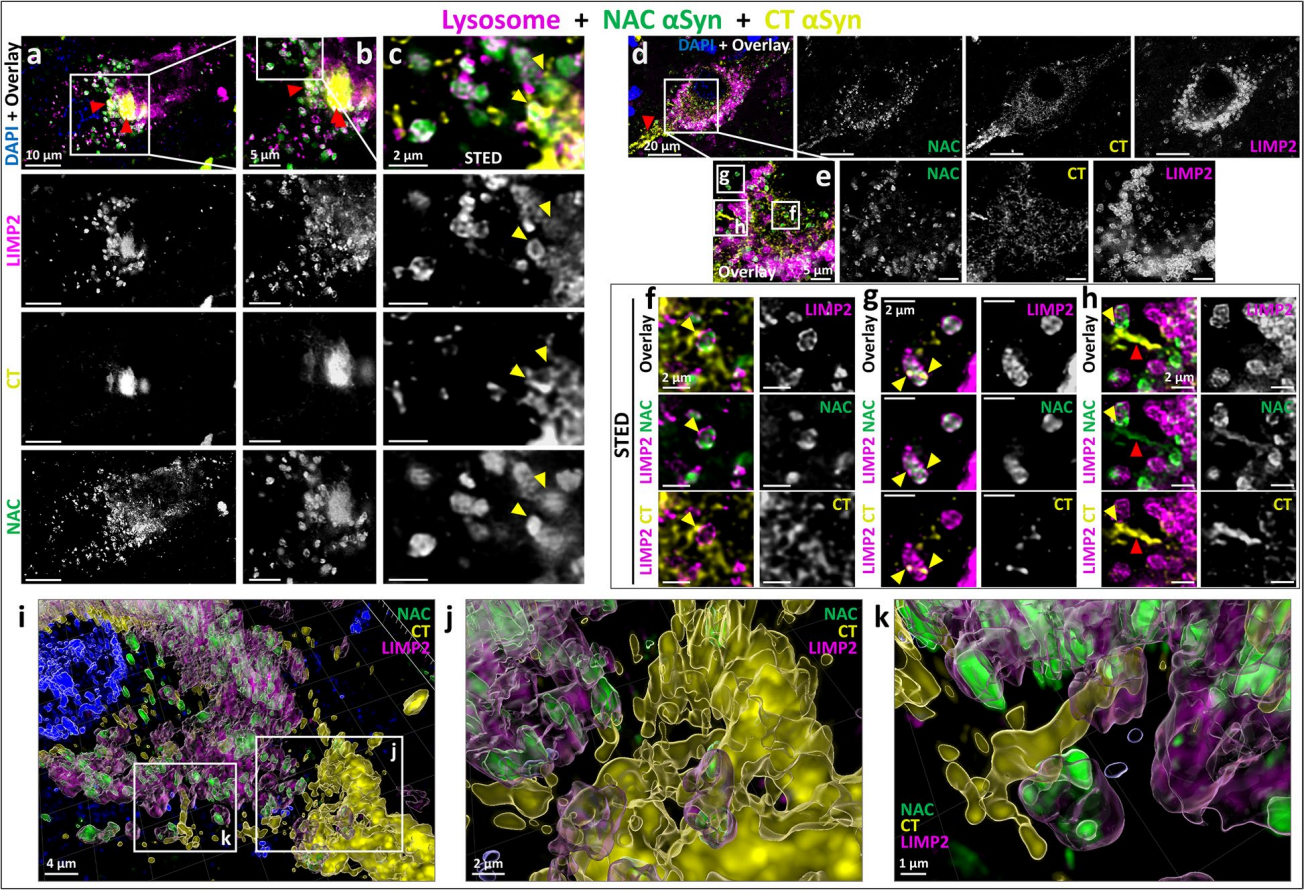
Distinct, co-existing somatic pools of pathological αSynuclein in dopaminergic neurons of the *substantia nigra* — **a-k** Representative deconvolved confocal (**a**,**b**,**d**,**e**,**i-k**) and stimulated emission depletion microscopy (STED) (**c**,**f-h**) multiplex immunofluorescence images and zoom-ins of dopaminergic *substantia nigra* neurons in Parkinson’s disease (PD) and PD with dementia *post-mortem* human brain stained for the lysosomal marker LIMP2 (magenta), NAC-targeting αSynuclein (αSyn) antibody (A15115A; green) and C-terminus (CT)-targeting antibody (5C1; yellow). The staining demonstrates the existence of two cellular pools of αSyn recognized by either CT-targeting or NAC-targeting antibodies with limited overlap in cytosolic macro-aggregates (red arrowheads). Yellow arrowheads indicate representative points of signal overlap at the lysosomal level. **f–h** Deconvolved STED images of the fields indicated in (**e**). **i-k** 3D surface rendering of a representative staining (**i**) and zoom-ins (**j**,**k**). DAPI is used for visualization of the nuclei (blue)

To further investigate the relationship between the reticular cytoplasmic network profiles observed with CT-targeting antibodies, and the dotted/ring-shaped morphologies observed with NT/NAC-targeting antibodies, we designed a MxIF experiment using the lysosomal markers LIMP2, NAC αSyn (clone A15115A), and CT αSyn (clone 5C1; Fig. 6). In pathological aggregates, we found a layered distribution of NT/NAC vs CT epitopes, which localized more centrally and peripherally, respectively, in larger inclusions (Suppl. Figure 5d-f), as previously observed [14]. Outside of the macro-aggregates, immunoreactivity patterns suggested the existence of two distinct pools of αSyn within each cell. One pool, identified by the CT-targeting αSyn staining, displayed a net-shaped cytosolic reticular network, as well as macro-aggregates and LBs, but no lysosomal staining (Fig. 6d,e). A second pool, identified by NT/NAC-targeting αSyn staining produced a dotted lysosomal pattern in the same cell, but no reticular network (Fig. 6a-e). The two immunopatterns showed colocalization only within and around macro-aggregates, such as LBs or amorphous aggregates, and in sporadic contact points. Closer analysis with STED microscopy showed that the CT-specific αSyn signal often localized around lysosomes but with limited colocalization (Fig. 6f-h). Points of signal overlap at the lysosomal membrane was occasionally observed between all three markers (Fig. 6c,f–h; yellow arrowheads). At times, this was accompanied by the formation of reticular aggregates in the cytoplasm, which were positive for both CT and NAC-specific signals (Fig. 6h). These results were confirmed by 3D surface reconstruction (Fig. 6i-k). Moreover, both pSer129 and CT-targeting antibodies revealed similar cytoplasmic reticular network morphologies, but no dotted vesicular patterns. Therefore, we tested whether these immunopatterns overlapped. MxIF experiments including pSer129 αSyn (clone EP1536Y), NAC αSyn (clone A15115A) and CT αSyn (clone 5C1) revealed overlapping reticular network patterns identified by both pSer129 and CT-targeting αSyn antibodies, indicating that this feature is detected by CT-directed αSyn antibodies, including those specific for pSer129 (Suppl. Figure 5, see Additional file 1).

## Discussion

An increasing number of studies have investigated the deposition of pathological αSyn in the neuronal soma in *post-mortem* human PD brain, revealing diverse αSyn morphologies depending on the antibody used [31, 44, 45, 58]. In our study, we identified the presence of two distinct intracellular αSyn pools in the soma of αSyn-positive DA nigral neurons. The first pool, detected by antibodies targeting the NT and NAC regions of αSyn, includes dotted and ring-shaped structures, as well as larger macro-aggregates in the cytosol. Notably, we demonstrated that ring-shaped αSyn is lysosome-associated, indicating a potential role of lysosomal αSyn accumulation in DA neurodegeneration in PD/PDD. The second pool, recognized by antibodies targeting the CT region, predominantly consists of non-lysosomal reticular networks, as well as larger macro-aggregates, but does not include the dotted and ring-shaped lysosomal morphologies. Our findings show that the observed morphological differences are driven by the location of the antibody epitope along the αSyn sequence (CT or non-CT), rather than the specific PTM targeted. Interestingly, while all analyzed αSyn-positive neurons displayed lysosomal αSyn, not all cells with lysosomal αSyn exhibited non-lysosomal αSyn morphologies. Our semi-quantitative analysis showed that cells displaying only dotted/ring-shaped lysosomal αSyn are most frequent at early Braak stages, with a gradual decline at later stages.

Our findings indicate that antibodies targeting the NAC and NT domains of αSyn detect a broader range of pathological morphologies compared to CT or pSer129-directed antibodies, consistent with previous reports [31]. While CT/pSer129 antibodies revealed reticular network patterns by confocal laser scanning microscopy (CLSM), NAC/NT antibodies did not. Notably, the reticular pattern was not visible in colorimetric IHC using bright-field imaging, which instead showed a diffuse cytoplasmic signal—possibly corresponding to the same structure. These discrepancies likely reflect differences in imaging resolution and signal amplification methods. The limited overlap between cytoplasmic structures labeled by NAC/NT versus CT/pSer129 antibodies suggests that these antibody groups identify distinct subcellular pools of αSyn. CT-directed antibodies, including those targeting pSer129, may miss critical species such as C-terminally truncated forms, underestimating the heterogeneity of αSyn pathology. This has important implications for neuropathological assessments, including Braak staging, and underscores the diagnostic value of incorporating NAC/NT-targeting antibodies to better capture the diversity of αSyn aggregates and their spreading patterns [14, 44, 58]. Moreover, given emerging evidence that lysosomal transfer may facilitate intercellular propagation of αSyn seeds [33, 34, 59], our results underscore the relevance of lysosomal αSyn species as potential therapeutic targets, for instance with the use of next-generation intracellular antibody delivery systems such as vector-mediated constructs, fusion proteins, or lipid nanoparticles [60–62].

Here, we provide a detailed characterization of the dotted morphologies detected by antibodies against the NT and NAC regions of αSyn. This morphology may match descriptions in previous reports [12, 45]. However, the extent of dotted αSyn could have been underappreciated thus far, in part owing to the traditional use of antibodies against the CT and due to difficulties in distinguishing DAB-stained cytoplasmic granules from neuromelanin in immunohistochemistry. Here, by using a blue chromogen and high-content fluorescent imaging, we demonstrated that this morphology is widespread and is associated with PD/PDD and iLBD, as this pattern was absent in control cases. The limited Syn1 signal observed in controls (Fig. 1v) was due to the presence of minimal non-somatic diffused signal, present in aged brain (possibly synaptic), not accounted for during *post-mortem* neuropathological examination. We found that dotted and ring-shaped αSyn morphologies colocalized with various lysosomal markers. This finding is consistent with previous publications, which have identified lysosomal αSyn using NT-targeting antibodies [31, 39]. Puska et al. observed similar morphological features and showed colocalization between αSyn and the lysosomal protease CatD [39]. In addition, a recent study in mouse primary cortical neurons, treated with αSyn pre-formed fibrils (PFFs), described similar morphologies, which were detected only using a subset of NT/NAC αSyn antibodies targeting aa 30 to aa 99 [58].

Given the central role of lysosomes in αSyn degradation and their frequent co-occurrence with macro-aggregates, it is plausible that the lysosomal αSyn observed in our study represents excessive and/or aggregated αSyn targeted for lysosomal degradation [29, 30, 63]. Lysosomal dysfunction has been identified as a prominent feature in preclinical models of PD [38, 57, 64–66], whereas mutations in the lysosomal enzyme GCase are among the most common genetic risk factors for PD [67]. Using super-resolution STED microscopy, we further demonstrated that lysosomal αSyn predominantly localized within the lysosomal lumen, with occasional contact points at the lysosomal membrane. This observation supports the hypothesis that pathological αSyn might be processed within lysosomes as part of its degradation pathway. Double immunogold analysis of lysosomal αSyn by EM in *post-mortem* human PD brain with 5G4 αSyn antibody (aa 46–53, NAC) and CatD antibody revealed dotted αSyn inclusions localized in the lumen of lysosomes, in accordance with our results [39]. Further investigations, including correlative light-electron microscopy (CLEM)-based analyses, are needed to validate these findings and explore the ultrastructure of these lysosomes and the conformation of lysosomal αSyn. As soluble αSyn has been proposed to also be degraded by the proteasome, it cannot be excluded that lysosomal αSyn accumulation might result from insufficient proteasomal degradation and subsequent targeting of undegraded protein to the lysosome via, for example, macroautophagy or chaperone-mediated autophagy [68–70]. Similarly, lysosomal αSyn accumulation may originate from internalized exogenous aggregated/fibrillar αSyn, which is targeted to lysosomes, where it could prime further protein aggregation.

CTT αSyn forms have been shown to represent a significant fraction of pathological αSyn species in PD brains and models [25, 58, 71]. Our findings suggest that lysosomal αSyn species are primarily truncated at the CT domain, while the CT of non-lysosomal αSyn aggregates remains accessible for antibody detection. Based on the combinations of antibodies evaluated in this study, the lysosomal-associated αSyn species appear to be cleaved between residue 92 and 107. The most likely truncation site lies between amino acids 99 and 103 (between epitopes of antibody clones Syn1 and 4B12; Fig. 5l). Notably, a well-characterized truncation site for αSyn is at residue 103 [71–73], which is believed to result from the action of lysosomal proteases such as asparagine endopeptidase (AEP) or Cathepsin L (CatL) [71]. In vitro and in vivo studies have shown that αSyn truncated at residue 103 not only displays a lower threshold for aggregation, but also accelerates the aggregation of full-length αSyn when co-polymerized, potentially initiating prion-like seeding in neurodegenerative diseases [71]. A scenario in which partially degraded αSyn accumulates within the confined lysosomal environment could be compatible with the results presented by us and others [58]. It remains unclear whether truncation per se is the primary trigger for toxicity, or if the accumulation of partially degraded αSyn within lysosomes — regardless of truncation status — initiates the formation of toxic species.

However, further studies are required to identify the exact truncated lysosomal αSyn species and to clarify their role in αSyn aggregation and pathology. Alternatively, these CT-lacking αSyn species may arise from alternative splicing of the *SNCA* gene. Splicing isoforms that produce αSyn variants lacking parts of the CT domain have been reported in the human brain [74, 75], warranting further investigation.

Our findings indicate that non-lysosomal αSyn might retain the CT domain and display a diverse PTM profile. This finding is consistent with studies in mouse αSyn PFF-treated primary neuronal mouse cultures, which also demonstrated two distinct αSyn pools (lysosomal and non-lysosomal) with different PTM profiles [58]. In this study, the authors demonstrate that PFFs are initially internalized into lysosomes where CT truncation occurs, followed by escape into the cytosol. In this model, PFFs induced the aggregation of endogenous αSyn, leading to the accumulation of CT-positive αSyn species with different truncation patterns and a more diverse PTM profile in the cytosol [58]. Notably, preventing αSyn cleavage did not affect the seeding capacity of PFFs [58]. Interestingly, the most prevalent lysosomal αSyn truncation in this study occurred at residue 114, differing from our findings in *post-mortem* human brain (between aa 92–107). This discrepancy may be due to species-specific differences in the αSyn sequence. Importantly, mouse αSyn contains a glycine (G) at position 103, whereas human αSyn has an asparagine (N) at this site, which could lead to the generation of different truncated species in mice compared to humans. Importantly, substantial truncation of αSyn at aa 103 was observed in human HEK293T cells treated with human αSyn PFFs, which co-aggregated with full-length αSyn in vitro [76].

Reticular network profiles were detected by CT- and pSer129-targeting antibodies, but minimally with NT/NAC antibodies. Our interpretation is that the differential antibody labeling observed in the cytosol is, at least in part, driven by epitope accessibility, which is influenced by the conformation and aggregation state of αSyn. Notably, NAC- and NT-directed antibodies do label cytoplasmic αSyn in certain structures, including macro-aggregates such as LBs and dense inclusions (e.g., Fig. 3a,b,d; Fig. 5a,b,d; Fig. 6a-e; Suppl. Figure 5), as well as reticular network structures (e.g., Fig. 6g,h, red arrowheads). In these aggregated morphologies, where αSyn is abundant, NAC and NT antibodies show some labeling, suggesting that part of the protein may retain accessible epitopes. To enhance epitope exposure, we applied multiple antigen retrieval protocols (Supplementary Table 2). Despite these measures, NT/NAC-directed antibodies consistently showed weak cytosolic labeling, suggesting that these structures might adopt a highly stable conformation resistant to aggressive antigen retrieval conditions. This is consistent with prior reports demonstrating the resistance of fibrillar αSyn to heat, extreme pH and proteolysis [45, 77, 78]. This persistent inaccessibility suggests that cytosolic aggregates may exist in a compact, aggregated conformation, whereas lysosomal αSyn may be less densely packed. The NAC and NT domains of αSyn have been shown to localize within the hydrophobic core of aggregated/fibrillar αSyn, and may be less accessible for binding [79–81]. Importantly, the acidic pH found within lysosomes has been shown to influence the conformation of αSyn [82].

Differential staining patterns between NT-targeting and pSer129-targeting (CT-targeting) αSyn antibodies have previously been demonstrated in human brain [31] and in PFF-treated mouse neurons [45]. In the latter study, epitope-dependent differences in antibody binding to recombinant αSyn species were observed by western blot, showing a trend for CT antibodies to preferentially detect high-molecular-weight (HMW) αSyn species, whereas NAC- and NT-targeting antibodies exhibited limited reactivity to HMW fibrils [45]. These results suggest that epitope masking due to aggregate conformation may be an important factor underlying the differential labeling patterns we observed. Further in vitro experiments are needed to validate this hypothesis.

**Fig. 7:**
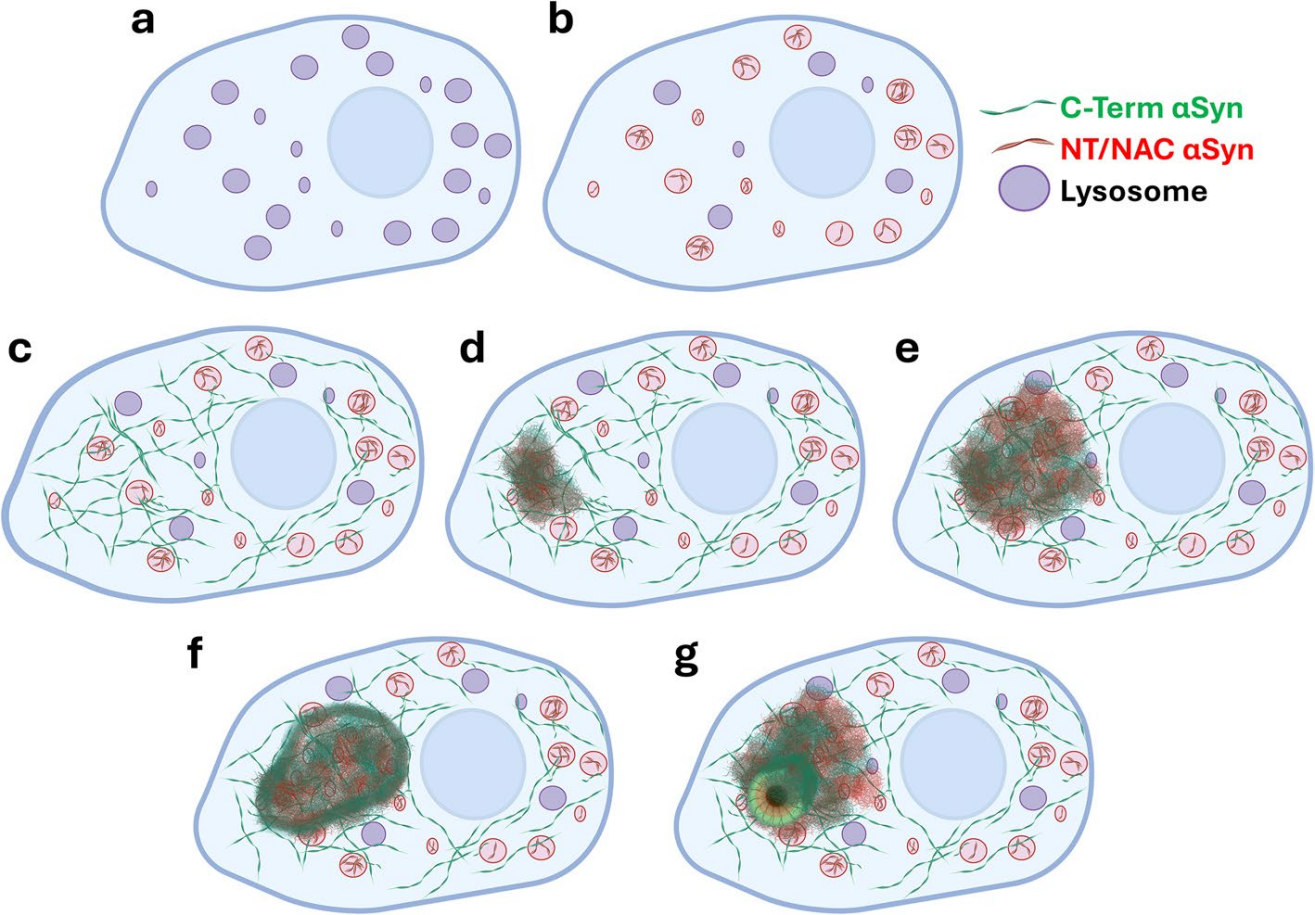
Hypothetical model of αSynuclein macro-aggregates and Lewy bodies formation in nigral PD neuronal soma — Representative graphical summary of hypothetical stages of neuronal αSynuclein (αSyn) accumulation in the *substantia nigra* of Parkinson’s disease (PD) and PD with dementia (PDD) patients showing lysosomes (purple), N-terminus (NT)/NAC-positive αSyn (red), and C-terminus (CT)-positive αSyn (green). **a** At first, cells show no detectable pathological αSyn deposition. **b** Initial accumulation is characterized by αSyn lacking the CT, which forms dotted and ring-shaped structures that localize predominantly at the lysosome. **c** A distinct network of reticular cytosolic αSyn structures emerges, containing CT-positive αSyn with minimal to no overlap with lysosomes, forming a separate intracellular αSyn pool. **d-g** Over time, neurons develop small amorphous inclusions and pale bodies (**d**,**e**), which progressively enlarge to form layered aggregates (**f**) and Lewy bodies (**g**)

In our investigation, all neurons with somatic macro-aggregates also exhibited lysosomal αSyn. Moreover, cells showing only lysosomal (dotted/ring-shaped) αSyn were most common at early Braak stages (1–4) and declined by stages 5–6. These observations support the hypothesis that lysosomal dotted/ring-shaped αSyn may represent an early stage of somatic deposition in SN dopaminergic neurons, preceding macro-aggregate formation. Together, we propose a hypothetical model in which, in the initial stages, neuronal somas first exhibit dotted and ring-shaped (NAC αSyn +/CT αSyn –) αSyn structures localized at lysosomes (Fig. 7b, Suppl. Figure 6b). No CT-positive cytosolic αSyn is observed at these early stages. In intermediate stages, neurons begin to display a diffuse reticular network of CT-positive αSyn with minimal colocalization with lysosomes, alongside persistent lysosomal CT-negative αSyn (Fig. 7c, Suppl. Figure 6c). The advanced stages are characterized by the formation of small and progressively larger amorphous inclusions within the cytosol (Fig. 7d,e, Suppl. Figure 6d,e). Finally, in the late stages, neurons display αSyn layering and nucleation (Fig. 7f, Suppl. Figure 6f), leading to the formation of intracellular LBs (Fig. 7g, Suppl. Figure 6g). Nonetheless, the *post-mortem* tissue used in this study is limited in its ability to establish a definitive pathological timeline; additional mechanistic studies in relevant cellular and animal models are necessary to validate this hypothesis.

Overall, our findings highlight the relevance of lysosome-associated αSyn in the PD brain. Understanding the roles of different subcellular pools of αSyn, how they interact, and what drives the transition between lysosomal and cytosolic compartments might provide important clues for elucidating the mechanisms underlying the progression of αSyn aggregation, and for developing therapeutic strategies aimed at preventing this key pathological event in PD.

## Supplementary Information

Additional file 1: Supplementary material.

Additional file 2: Supplementary video 1: 3D surface rendering video of deconvolved confocal multiplex immunofluorescence images of a representative nigral dopaminergic neuronal soma from a Parkinson’s disease case. The tissue was stained for αSynuclein (green; Syn1, aa 91-99) and the lysosomal marker LIMP2 (red). aa = amino acid.

Additional file 3: Supplementary video 2: 3D surface rendering video of deconvolved confocal multiplex immunofluorescence images of a representative nigral dopaminergic neuronal soma from a Parkinson’s disease case. The tissue was stained for αSynuclein (green; Syn1, aa 91-99) and the lysosomal marker Cathepsin D (red). aa = amino acid.

Additional file 4: Supplementary video 3: 3D surface rendering video of deconvolved confocal multiplex immunofluorescence images of a representative nigral dopaminergic neuronal soma from a Parkinson’s disease case. The tissue was stained for αSynuclein (green; Syn1, aa 91-99) and the lysosomal marker β-Glucocerebrosidase (red). aa = amino acid.

Additional file 5: Supplementary video 4: 3D surface rendering video of deconvolved confocal multiplex immunofluorescence images of a representative nigral dopaminergic neuronal soma from a Parkinson’s disease case. The tissue was stained with lysosomal marker antibody LIMP2 (magenta), NAC-targeting αSynuclein (αSyn) antibody (A15115A; green), and C-terminus αSyn targeting antibody (5C1; yellow). DAPI is used for visualization of the nuclei (blue). aa = amino acid.

## Data Availability

No datasets were generated or analysed during the current study.
