# Supplementary material for: C-terminus-dependent detection of lysosomal alpha-synuclein in nigral Parkinson’s disease human brain neurons
Source: Mol Neurodegener. 2025 Oct 31;20:116. doi: 10.1186/s13024-025-00884-3 (PMC12577314; doi:10.1186/s13024-025-00884-3)
Supplement: Supplementary file 1 — Additional file 1: Supplementary material. [file 13024_2025_884_MOESM1_ESM.pdf]

Supplementary Material

C-terminus-dependent detection of lysosomal alpha-synuclein in nigral Parkinson's disease human brain

Martino L Morella[1,2], Bana Al Khayrat[1,2], Tim E Moors[1,2], Lisanne in 't Veld[1,2], Irene Frigerio[1,2], Bram L van der Gaag[1,2], Vinod Udayar[3], Wilma DJ van de Berg[1,2, †]

Affiliations:

[1] Section Clinical Neuroanatomy and Biobanking, Department of Anatomy and Neurosciences, Amsterdam UMC, Vrije University Amsterdam, Amsterdam, The Netherlands.

[2] Amsterdam Neuroscience, program Neurodegeneration, Amsterdam, The Netherlands

[3] Roche Pharma Research and Early Development; Neuroscience and Rare Diseases Discovery and Translational Area; Roche Innovation Center, F. Hoffmann-La Roche Ltd, Grenzacherstrasse 124, 4070 Basel, Switzerland.

Supplementary figures

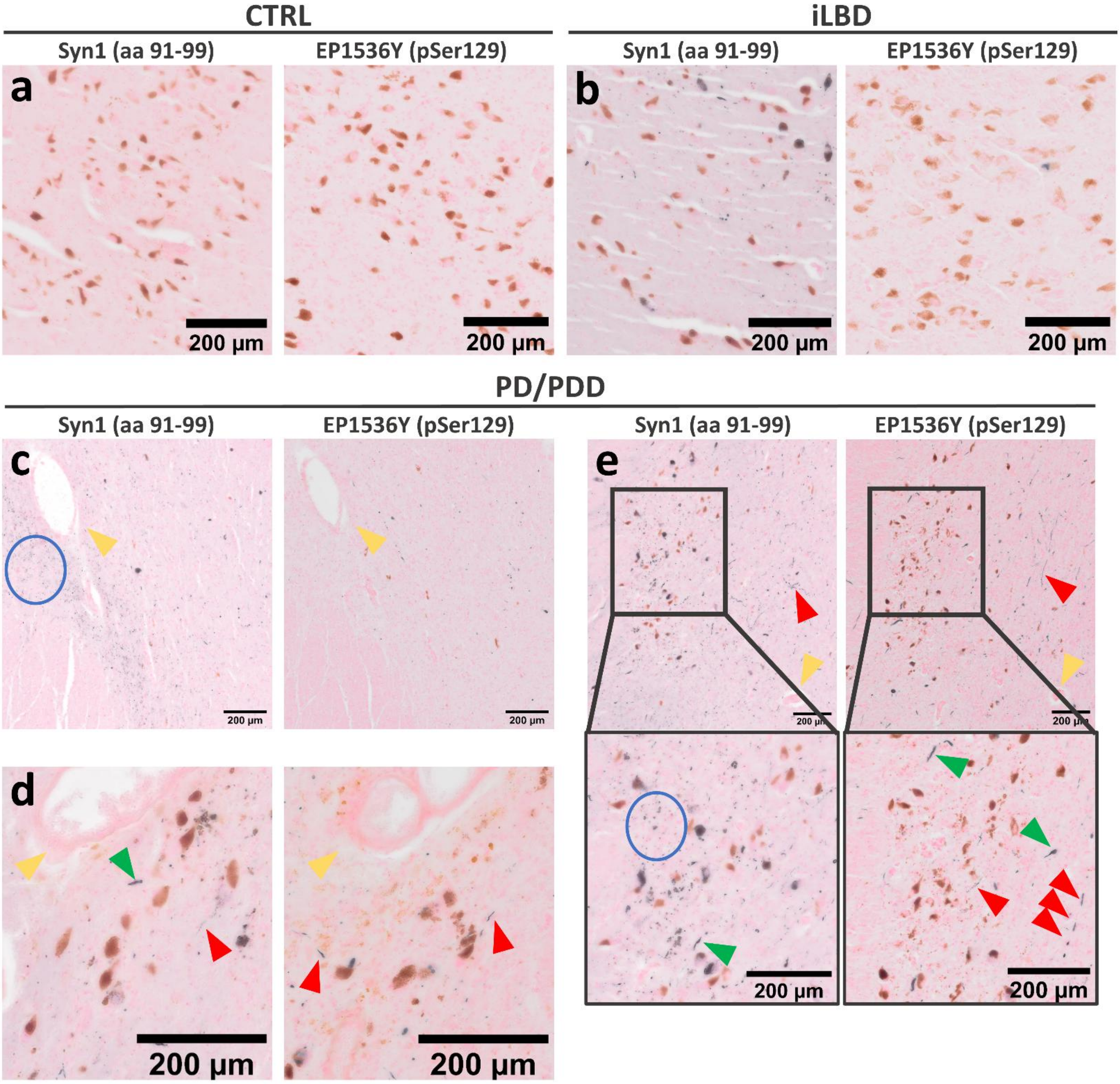

Supplementary Figure 1: Syn1 and pSer129 αSynuclein immunostaining patterns in sequential sections of *substantia nigra* from *post-mortem* human brain of controls, iLBD and PD/PDD cases.

a-e: Representative immunohistochemistry images of αSynuclein (αSyn; blue staining) with either Syn1 antibody (αSyn aa 91-99; left panels) or EP1536Y antibody (Serine 129-phosphotylated αSyn, pSer129; right panels) in the *substantia nigra* of *post-mortem* human brain from controls (CTRL; **a**), incidental Lewy body disease (iLBD; **b**), Parkinson's disease (PD), and PD with dementia (PDD) cases (PD/PDD; **c-e**). Zoom-ins of the indicated areas are presented in **e** (lower panels). The stainings with the two antibodies were performed on sequential 20 µm sections demonstrating the difference in the

morphologies recognized and in the amount of positive area between the two αSyn antibodies. Yellow arrowheads indicate preserved landmarks between sequential tissue sections of the same case. Red arrowheads indicate representative Lewy neurites and dysmorphic neurites. Green arrowheads indicate representative bulgy neurites. Blue circles indicate representative areas of extrasomatic dotted staining. aa = amino acid.

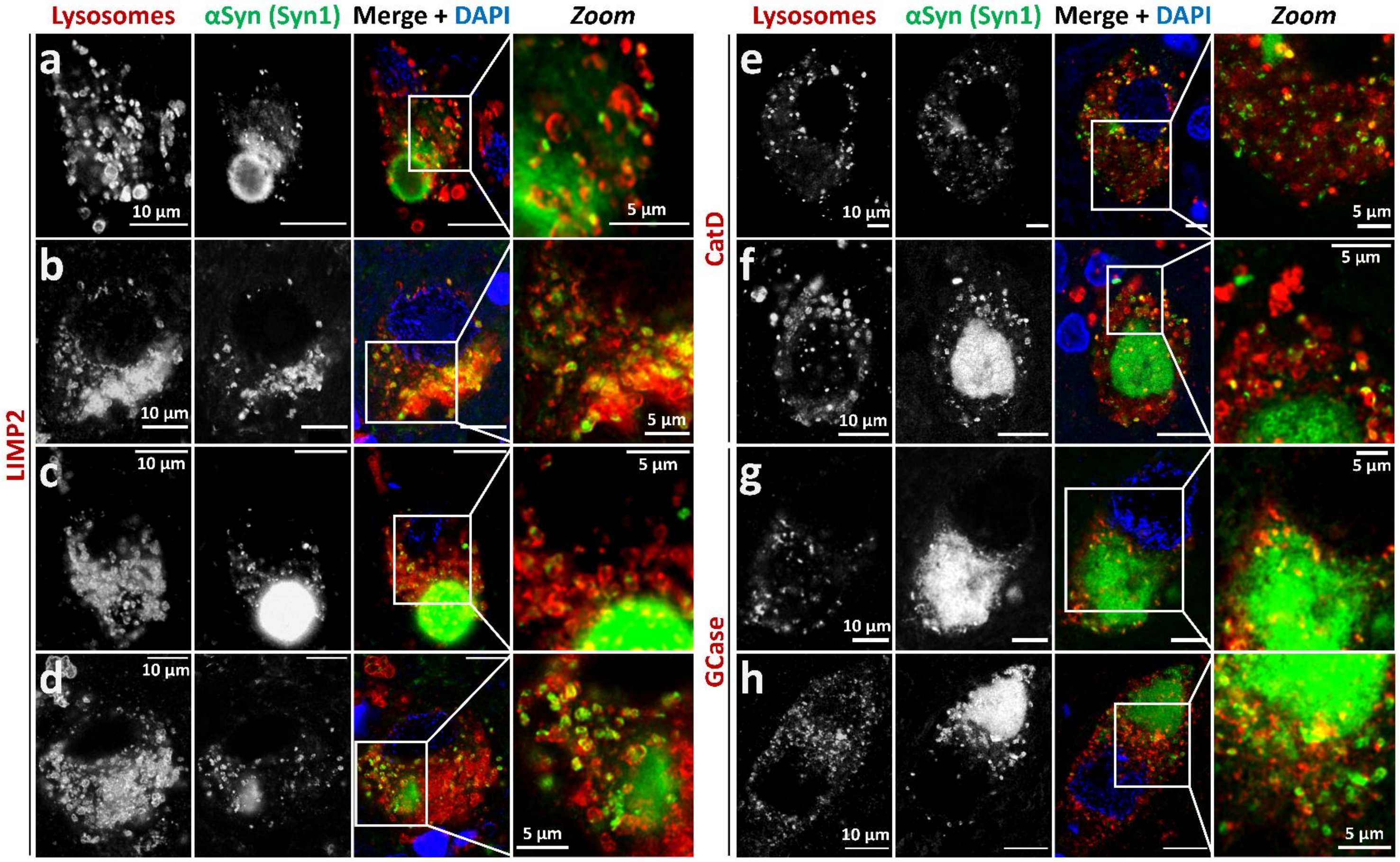

Supplementary Figure 2: Additional representative images of lysosomal localization of ring-shaped αSynuclein in the somas of human dopaminergic *substantia nigra* neurons.
a-h: Representative deconvolved confocal multiplex immunofluorescence images and magnifications (right panels) of αSynuclein (αSyn; green, Syn1 aa 91-99) in nigral dopaminergic neurons from Parkinson's disease (PD) and PD with dementia *post-mortem* human brain showing the localization of ring-shaped somatic αSyn morphology with lysosomal markers (red throughout). **a-d:** Colocalization with LIMP2. **e-f:** Colocalization with Cathepsin D (CatD). **g-h:** Colocalization with β-Glucocerebrosidase (GCase). DAPI is used for the visualization of the nuclei (blue). aa = amino acid.

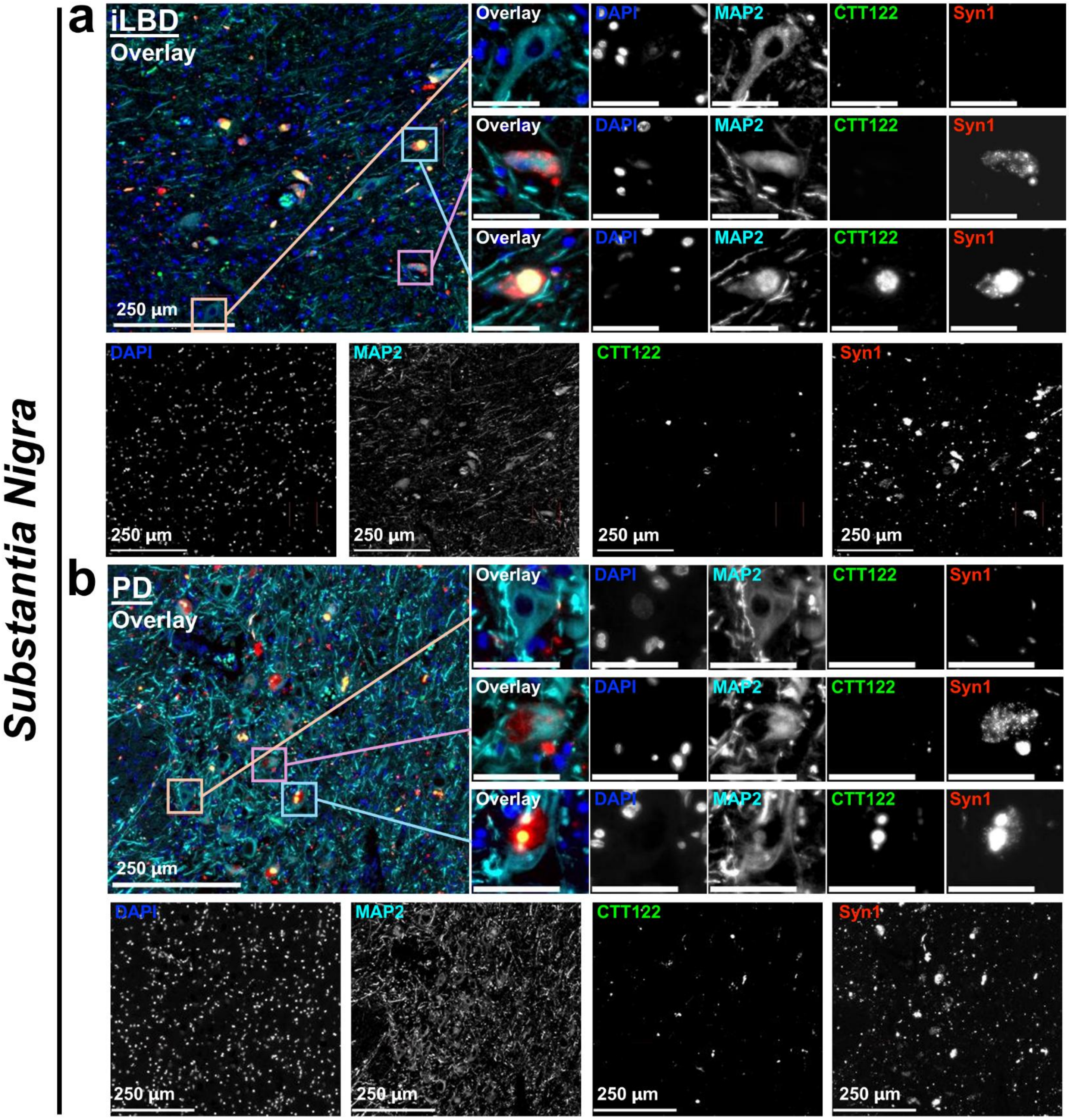

Supplementary Figure 3: Representative high-content imaging of aa 91-99 and CTT122-targeting αSynuclein immunostaining in the *substantia nigra*.

a-b: Representative high-content multiplex immunofluorescence widefield images and magnifications (highlighted cells; right panels) of *post-mortem substantia nigra* tissue stained with DAPI (blue), neuronal marker MAP2 (cyan), aa 91-99 αSynuclein (αSyn)-targeting antibody (Syn1, red) and aa 122 C-terminus truncated αSyn-targeting antibody (CTT122, green) of an incidental Lewy body disease (iLBD) case (**a**) and Parkinson's disease (PD) case (**b**). Scale bars = 50 µm, unless differently indicated. aa = amino acid.

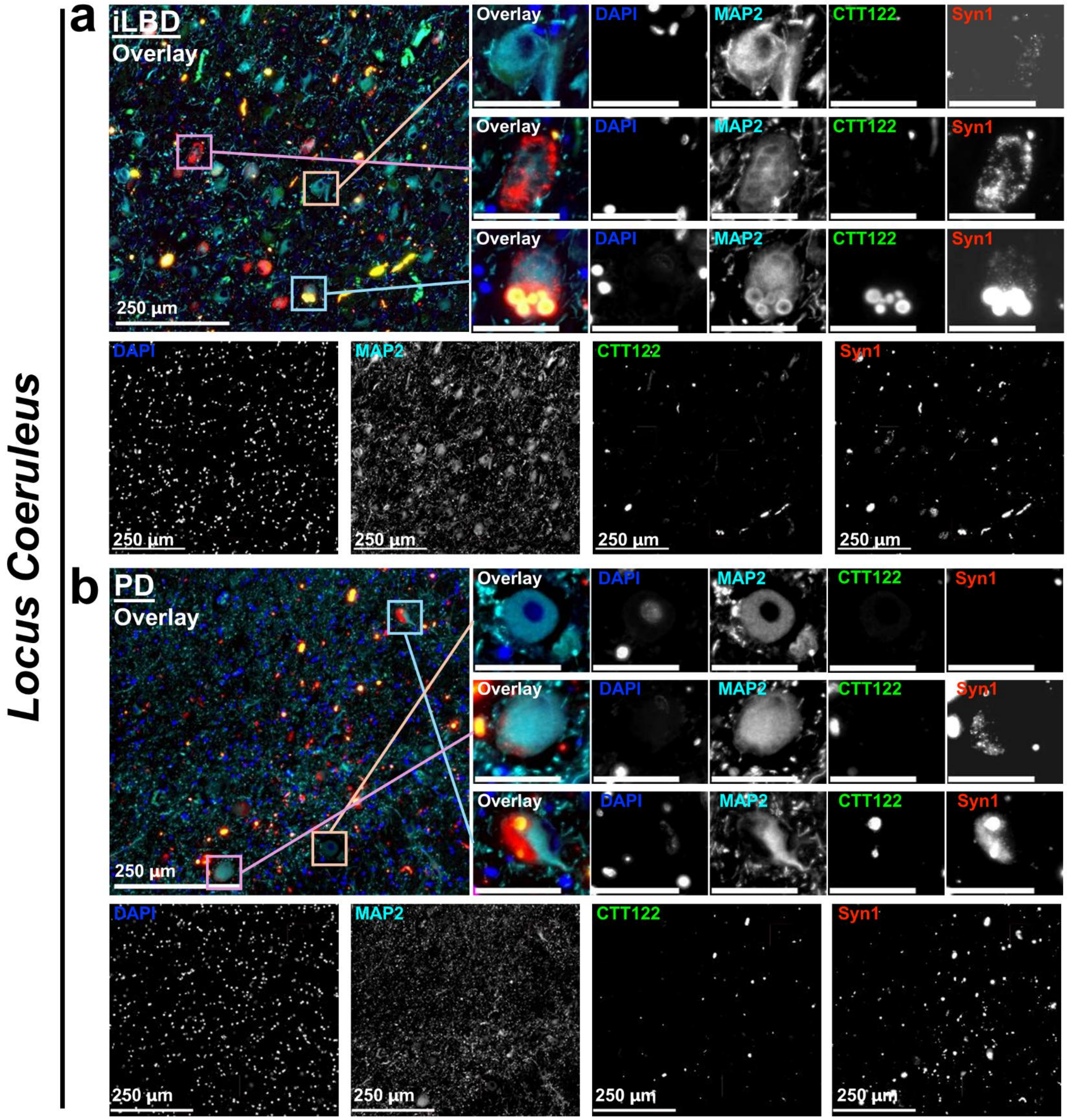

Supplementary Figure 4: Representative high-content imaging of aa 91-99 and CTT122-targeting αSynuclein immunostaining in the *locus coeruleus*.

a-b: Representative high-content multiplex immunofluorescence widefield images and magnifications (highlighted cells; right panels) of *post-mortem locus coeruleus* tissue stained with DAPI (blue), neuronal marker MAP2 (cyan), aa 91-99 αSynuclein (αSyn)-targeting antibody (Syn1, red) and aa 122 C-terminus truncated αSyn-targeting antibody (CTT122, green) of an incidental Lewy body disease (iLBD) case (**a**) and Parkinson's disease (PD) case (**b**). Scale bars = 50 µm, unless differently indicated. aa = amino acid.

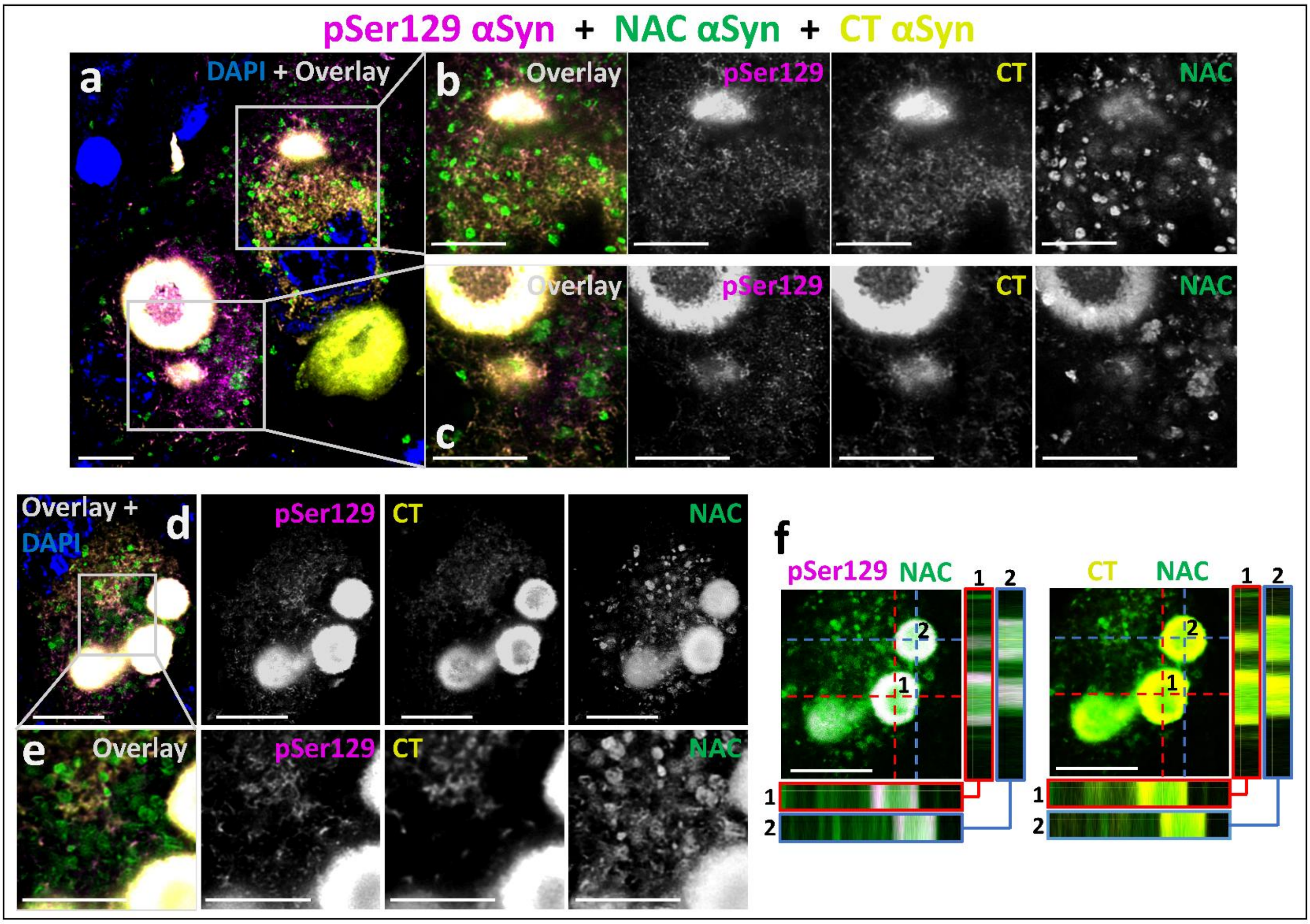

Supplementary Figure 5: Comparison of αSynuclein immunopatterns obtained with NAC, C-terminus and pSer129-targeting antibodies in the soma of human dopaminergic *substantia nigra* neurons.

a-e: Representative deconvolved confocal laser scanning microscopy multiplex immunofluorescence images and zoom-ins of immunostaining with the NAC-targeting αSynuclein (αSyn) antibody (A15115A; green), C-terminus (CT) targeting antibody (5C1; yellow) and an antibody targeting Ser129-phosphorylated (pSer129) αSyn (EP1536Y; purple) in dopaminergic neurons of the *substantia nigra* in *post-mortem* brain of Parkinson's disease (PD) and PD with dementia cases. pSer129 and CT-specific signals are largely overlapping, while NAC-specific signal recognizes a separate pool of αSyn. **f**: Orthogonal views of intracellular Lewy bodies (LBs) from (**d**,**e**) showing the layering of αSyn in LBs as identified by the multiplexed use of NAC-targeting and CT/pSer129-targeting antibodies. DAPI is used for visualization of the nuclei (blue). Scale bars = 10 μm. aa = amino acid.

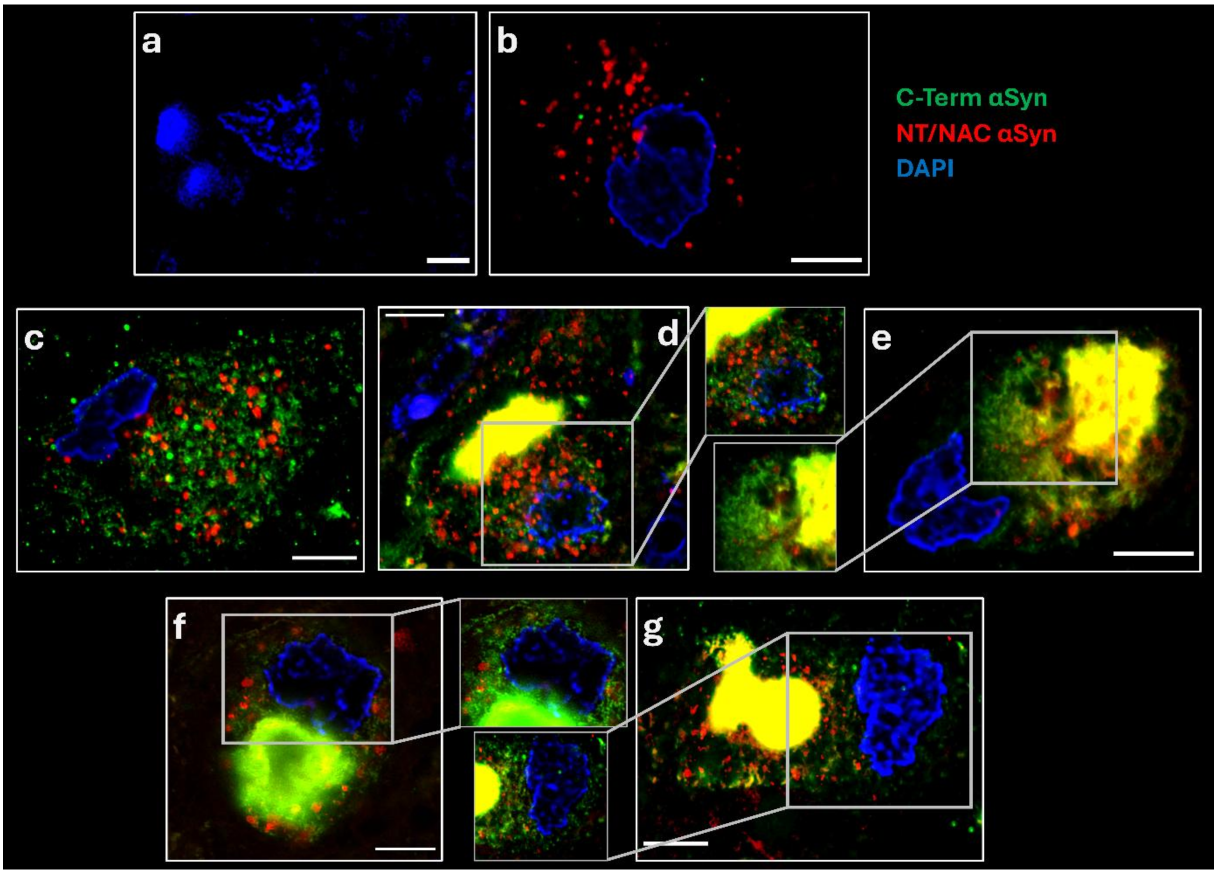

Supplementary Figure 6: Hypothetical model of αSynuclein macro-aggregates and Lewy bodies formation in nigral PD/PDD neuronal soma.

Representative deconvolved confocal multiplex immunofluorescence images of the hypothetical αSynuclein (αSyn) accumulation stages observed in the soma of dopaminergic neurons of the *substantia nigra* in Parkinson's disease (PD) and PD with dementia patients when staining for NAC αSyn (aa 80-96, clone A15115A; red) and C-terminus (CT) αSyn (aa 118-126, clone 5C1; green). The images are a maximum-intensity projection of z-stack representative of the whole cell soma obtained in 30 µm-thick FFPE tissue sections. Overexposed zoom-ins are presented as indicated to show the presence of cytoplasmic αSyn reticular network besides the macro-aggregates, when present. **a:** Cells showing no αSyn deposition. **b:** Cells showing dotted and ring-shaped CT-negative (NT/NAC αSyn+, CT αSyn-) αSyn that localizes at the lysosomes. **c:** Besides lysosomal CT-negative αSyn, neurons show a reticular network of CT-positive αSyn with little to no colocalization with lysosomes. **d-e:** Neurons develop small (**d**) and progressively larger (**e**) amorphous inclusions. **f-g**: Neurons showing initial layering and a nucleation center (**f**), followed by the formation of intracellular Lewy bodies (**g**). DAPI is used for visualization of the nuclei (blue). Scale bars = 10 µm. aa = amino acid.

Supplementary tables

Supplementary Table 1: Group characteristics of controls, iLBD and PD/PDD cases in the present study.

	**Controls (N=8)**	**iLBD (N=30)**	**PD/PDD (N=18)**
**Age of death (years; mean ± SD)**	78 ± 3.2	85 ± 8.1	78 ± 6.9
**Sex (M/F)**	4/4	9/21	12/6
**Postmortem delay (hours; mean ± SD)**	7.20 ± 2.31	7.17 ± 1.99	6.29 ± 1.48
**Braak Lewy Body stage [1]**	0 (8)	1-2-3-4-5 (6/3/11/9/1)	4-5-6 (1/9/8)
**Braak stage for Neurofibrillary Tangles [2]**	0-1-3 (1/4/3)	0-1-2-3 (1/13/7/9)	0-1-2-3 (1/8/4/5)

Supplementary Table 2: Detailed demographic of the cases included in this study. Braak αSyn stage according to [1]. Braak stage for Neurofibrillary Tangles (Braak NFT stage) according to [2]. PD/PDD = Parkinson's disease (PD) or PD with dementia (PDD); iLBD = incidental Lewy body disease; CTRL = control. M = Male; F = Female. PMD = post-mortem delay.

ID	Group	Age	Sex	Braak αSyn stage	Braak NFT stage	PMD (min)
**01**	PD/PDD	83	F	5	3	310
**02**	iLBD	91	F	3	1	310
**03**	PD/PDD	86	F	5	1	360
**04**	iLBD	93	F	3	2	240
**05**	iLBD	98	M	3	2	390
**06**	PD/PDD	81	F	5	0	365
**07**	iLBD	80	F	2	1	415
**08**	PD/PDD	84	M	5	1	540
**09**	iLBD	80	F	5	1	300
**10**	PD/PDD	83	M	5	1	290
**11**	PD/PDD	74	M	5	1	405
**12**	PD/PDD	70	M	5	1	315
**13**	iLBD	70	M	4	0	375
**14**	iLBD	89	F	4	3	370
**15**	PD/PDD	77	M	6	3	350
**16**	iLBD	82	M	0	1	350
**17**	iLBD	89	F	4	2	285
**18**	PD/PDD	65	M	6	1	265
**19**	iLBD	87	F	4	1	420
**20**	CTRL	75	F	0	1	325
**21**	iLBD	95	F	3	1	315
**22**	iLBD	82	F	2	3	400
**23**	PD/PDD	86	M	6	3	350
**24**	iLBD	85	F	4	3	400
**25**	PD/PDD	86	M	4	2	445
**26**	iLBD	89	M	1	1	410
**27**	CTRL	75	M	0	1	430
**28**	iLBD	95	M	1	2	435
**29**	PD/PDD	75	F	6	3	375
**30**	PD/PDD	71	F	6	2	545
**31**	PD/PDD	69	M	6	1	410
**32**	iLBD	92	F	1	3	395
**33**	PD/PDD	83	M	6	2	310
**34**	CTRL	82	F	0	1	585
**35**	PD/PDD	88	F	5	2	335
**36**	iLBD	92	F	3	1	465
**37**	PD/PDD	70	M	6	3	270
**38**	PD/PDD	80	M	5	1	555

**39**	iLBD	91	F	3	3	570
**40**	iLBD	84	F	3	3	470
**41**	iLBD	96	F	4	2	430
**42**	iLBD	89	F	3	3	290
**43**	iLBD	94	M	1	3	265
**44**	iLBD	82	M	3	2	495
**45**	iLBD	72	M	4	1	505
**46**	CTRL	78	M	0	0	725
**47**	iLBD	76	F	3	2	540
**48**	iLBD	86	F	4	3	400
**49**	CTRL	84	F	0	3	300
**50**	CTRL	69	F	1	1	765
**51**	CTRL	77	M	0	3	350
**52**	iLBD	82	M	4	1	450
**53**	iLBD	72	M	3	1	705
**54**	iLBD	78	F	1	1	600
**55**	CTRL	78	F	0	3	390
**56**	iLBD	77	F	2	1	505

Supplementary Table 3: Details of all primary antibodies used in the present study. All antibodies were tested for immunohistochemistry with the following antigen retrieval (AR) methods: 30 min Heat Induced Epitope Retrieval (HIER) in 10 mM Citrate Buffer pH 7,0 (CB-HIER); 30 min HIER in 10mM Tris-EDTA buffer pH 9.0 (EDTA-HIER); 10 min in 100% Formic Acid (FA); CB-HIER + FA; EDTA-HIER + FA; 10 min 1 units/mL Proteinase K in Tris-EDTA buffer pH 8.0 at 37°C. Successful AR methods used in this study are reported for each antibody.

Target Protein	Clone Name	Ref. nr.	RRID	Source	Epitope	Host (clonality)	Dilution (µg/ml)	Antigen Retrieval IHC
**αSyn**	A15110D (LASH-BL 34-45)	849102	AB_2650701	Biolegend	aa 34-45	Mouse (monoclonal)	1:1000 (0.5)	CB-HIER + FA
**αSyn**	A15119B (LASH-BL pY39)	849201	AB_2650704	Biolegend	pTyr39	Mouse (monoclonal)	1:100 (5)	CB-HIER + FA
**αSyn**	5G4	MABN389	AB_2716647	Merck Millipore	aa 46-53	Mouse (monoclonal)	1:800 (2.5)	CB-HIER + FA
**αSyn**	A15115A	848302	AB_2650688	Biolegend	aa 80-96	Mouse (monoclonal)	1:1000 (0.5)	CB-HIER + FA
**αSyn**	A15115A - Biotinylated	848306	AB_2650688	Biolegend	aa 80-96	Mouse (monoclonal)	1:50 (10)	CB-HIER + FA
**αSyn**	Syn1 (Clone 42)	610786	AB_398107	BD Biosciences	aa 91-99	Mouse (monoclonal)	1:200 (1.25) STED: 1:100 (2.5)	CB-HIER + FA
**αSyn**	4B12	807801	AB_2564730	Biolegend	aa 103-108	Mouse (monoclonal)	1:2500 (0.4)	CB-HIER + FA
**αSyn**	LB509	ab27766	AB_727020	Abcam	aa 115-122	Mouse (monoclonal)	1:300 (3.33)	CB-HIER
**αSyn**	5C1	n.a.	n.a.	Prothena (Gift)	aa 118-126	Mouse (monoclonal)	1:4000 (0.84)	CB-HIER or CB-HIER + FA
**αSyn**	MJFR1	ab138501	AB_2537217	Abcam	aa 118-123	Rabbit (polyclonal)	1:200 (0.5)	EDTA-HIER

**αSyn**	asyn-131	n.a.	n.a.	Roche (Gift)	CTT119	Rabbit (polyclonal)	1:1000 (2)	EDTA-HIER
**αSyn**	Syn105	n.a.	n.a.	Prothena (Gift)	CTT122	Rabbit (polyclonal)	1:1000 (0.5)	CB-HIER
**αSyn**	A15127A	848402	AB_2650690	Biolegend	CTT122	Mouse (monoclonal)	1:100 (5)	EDTA-HIER + FA
**αSyn**	11A5	n.a.	n.a.	Prothena (Gift)	pSer129	Mouse (monoclonal)	1:20000 (0,3)	CB-HIER or CB-HIER + FA
**αSyn**	EP1536Y	ab51253	AB_1193226	Abcam	pSer129	Rabbit (polyclonal)	1:300 (7.73)	EDTA-HIER or CB-HIER + FA
**LIMP2 (SCARB2)**	n.a.	NB400-129	AB_2301298	Novus Biologicals	C-terminus	Rabbit (polyclonal)	1:600 (n.a.) STED: 1:400 (n.a.)	CB-HIER or CB-HIER + FA or EDTA-HIER + FA
**Cathepsin D**	EPR3057Y	ab75852	AB_1523267	Abcam	n.a.	Rabbit (polyclonal)	1:200 (0.93)	CB-HIER + FA
**GCase (GBA)**	EPR5143(3)	ab128879	AB_11144121	Abcam	n.a.	Rabbit (recombinant monoclonal)	1:100 (2.24)	CB-HIER + FA
**MAP2**	n.a.	NB300-213	AB_2138178	Novus Biologicals	n.a.	Chicken (polyclonal)	1:400 (n.a.)	CB-HIER or CB-HIER + FA or EDTA-HIER + FA

Supplementary Table 4: Details of the secondary antibodies used in the present study. CLSM = Confocal Laser Scanning Microscopy , MxIF = Multiplexed Immunofluorescence, STED = Stimulated Emission Depletion Microscopy.

**Host Clonality**	**Target Species**	**Ref. nr.**	**RRID**	**Source**	**Conjugate**	**Dilution (µg/ml)**	**Application**
Goat (polyclonal)	Rabbit	A-21245	AB_141775	Thermo Fisher Scientific	Alexa Fluor 647	1:200 (10)	CLSM
Goat (polyclonal)	Mouse	A-11003	AB_141370	Thermo Fisher Scientific	Alexa Fluor 546	1:200 (10)	CLSM
Donkey (polyclonal)	Mouse	A32787	AB_2762830	Thermo Fisher Scientific	Alexa Fluor 647 Plus	1:200 (10)	CLSM + MxIF-CLSM
Donkey (polyclonal)	Rabbit	A10040	AB_2534016	Thermo Fisher Scientific	Alexa Fluor 546	1:200 (10)	CLSM
Donkey (polyclonal)	Rabbit	A-21206	AB_2535792	Thermo Fisher Scientific	Alexa Fluor 488	1:200 (10)	MxIF-CLSM
[Streptavidin]	-	S11225	AB_2532130	Thermo Fisher Scientific	Alexa Fluor 546	1:200 (5)	CLSM + MxIF-CLSM
Goat (polyclonal)	Rabbit	ST635P-1002	AB_2893229	Abberior	STAR 635P	1:100 (10)	STED
Donkey (polyclonal)	Mouse	A-21202	AB_141607	Thermo Fisher Scientific	Alexa Fluor 488	1:100 (20)	STED

Supplementary Table 5: Categorization of somatic αSynuclein morphologies observed in dopaminergic *substantia nigra* PD/PDD and iLBD neurons with Syn1 antibody.
Representative immunohistochemistry images (right panels) of defined somatic αSynuclein (αSyn) morphologies (**a-e**) in dopaminergic *substantia nigra* neurons observed in incidental Lewy body disease, Parkinson's disease (PD) and PD with dementia *post-mortem* human brain tissue. The definition of the morphologies is based on Syn1 immunostaining (blue staining) of 20 µm-thick tissue sections. Scale bars = 10 µm.

**Somatic morphology**	**Description**	**Representative images**
**a** – Negative	No detectable αSyn staining	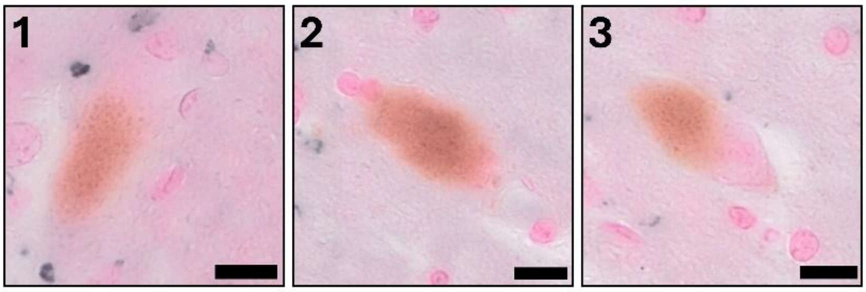
**b** – Dotted / Ring–shaped (DR)	Dotted / ring–shaped (DR) αSyn morphologies	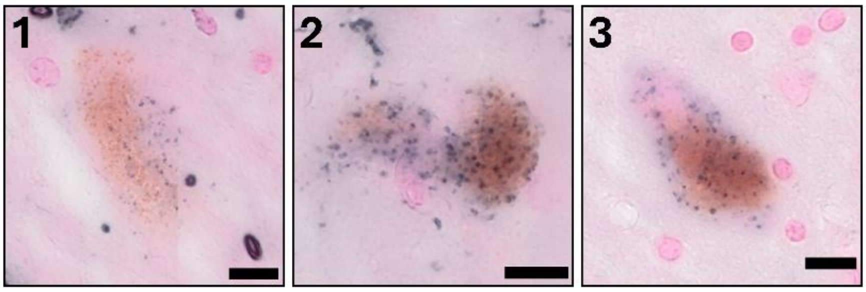
**c** – (DR +) Dense inclusion	DR + additional presence of an area of centralized dense cytoplasmic staining	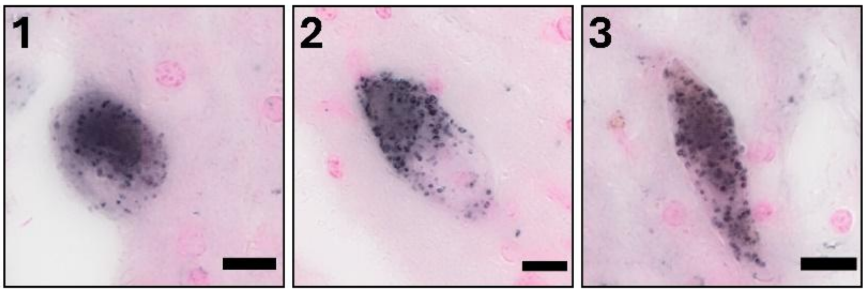
**d** – (DR +) Small / incomplete layered aggregate(s)	DR + additional presence of (incomplete) dark layer surrounding an area of diffused dense staining (diameter < 12 µm)	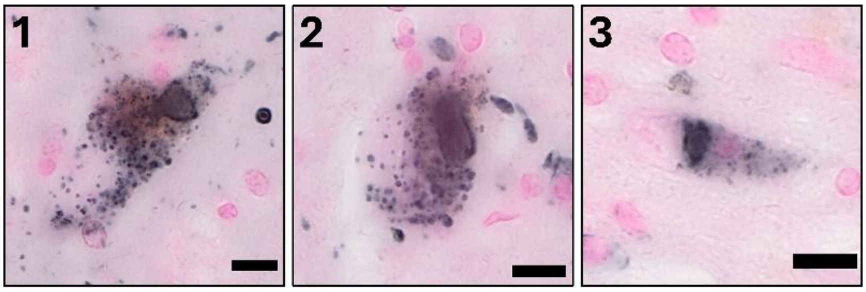

**e** – (DR +) Mature layered aggregate(s)	DR + big and dense aggregate surrounded by fully-formed layering (diameter > 12 μm)	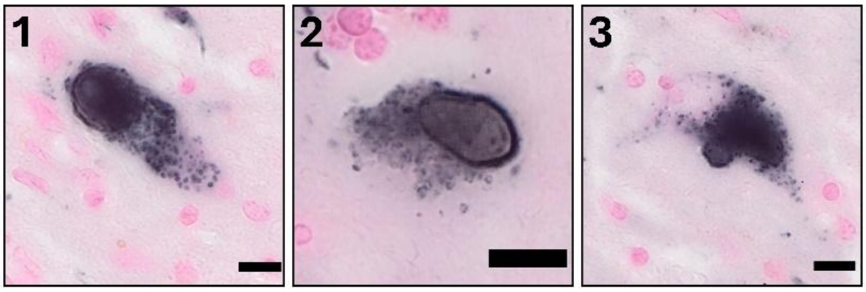

Supplementary Table 6: Categorization of somatic αSynuclein morphologies observed in dopaminergic *substantia nigra* PD/PDD and iLBD neurons with pSer129 antibody. Representative immunohistochemistry images (right panels) of defined somatic αSynuclein (αSyn) morphologies (**a-e**) in dopaminergic *substantia nigra* neurons observed in incidental Lewy body disease, Parkinson's disease (PD) and PD with dementia *post-mortem* human brain tissue. The definition of the morphologies is based on pSer129 (EP1536Y antibody) immunostaining (blue staining) of 20 µm-thick tissue. Scale bars = 10 µm.

**Somatic morphology**	**Description**	**Representative images**
**a** – Negative	No detectable αSyn staining	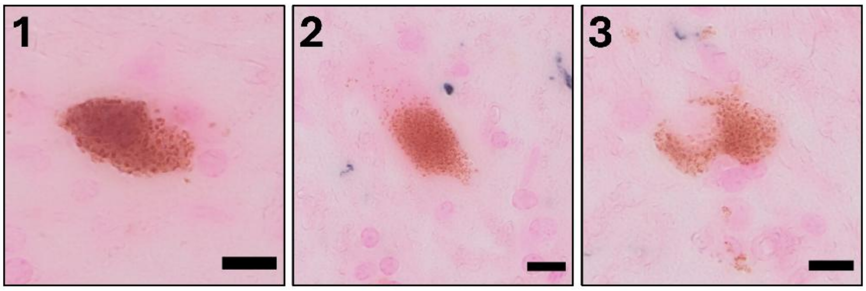
**b** – Diffused	Diffused cytoplasmic staining	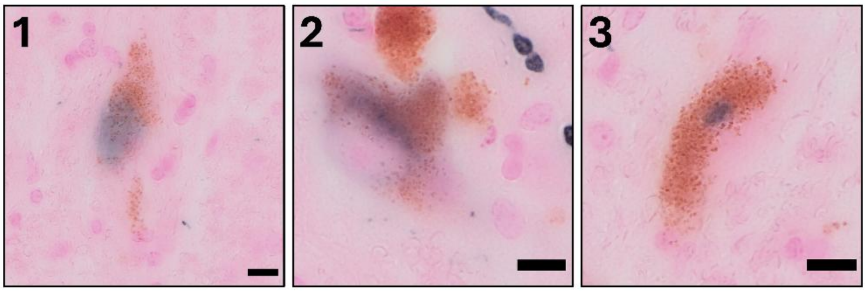
**c** – Dense inclusion	Additional presence of denser staining	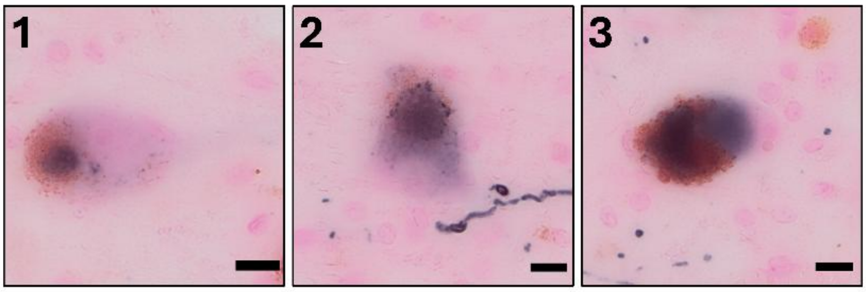
**d** – Small / incomplete layered aggregate(s)	Additional presence of partial dark layering surrounding an area of denser staining	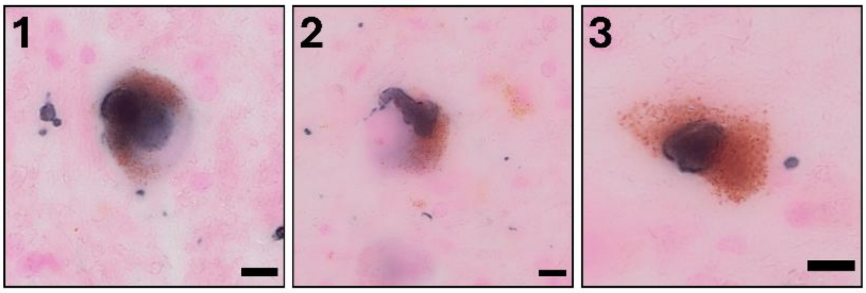

**e** – Mature layered aggregate(s)	Dense dark aggregate surrounded by extensive layering	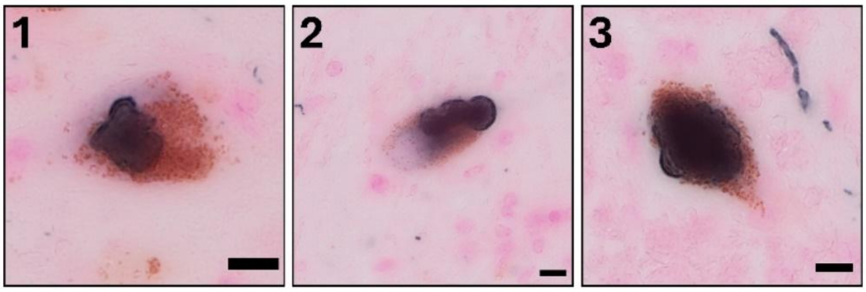

References

1. Alafuzoff I, Ince PG, Arzberger T, Al-Sarraj S, Bell J, Bodi I, Bogdanovic N, Bugiani O, Ferrer I, Gelpi E, et al: **Staging/typing of Lewy body related alpha-synuclein pathology: a study of the BrainNet Europe Consortium.** *Acta Neuropathol* 2009, **117:**635-652.
2. Montine TJ, Phelps CH, Beach TG, Bigio EH, Cairns NJ, Dickson DW, Duyckaerts C, Frosch MP, Masliah E, Mirra SS, et al: **National Institute on Aging-Alzheimer's Association guidelines for the neuropathologic assessment of Alzheimer's disease: a practical approach.** *Acta Neuropathol* 2012, **123:**1-11.
